# Tai Chi and Qigong Practices for Chronic Heart Failure: A Systematic Review and Meta-Analysis of Randomized Controlled Trials

**DOI:** 10.1155/2020/2034625

**Published:** 2020-12-14

**Authors:** Xiankun Chen, Gianluigi Savarese, Yiyi Cai, Liuling Ma, Cecilia Stålsby Lundborg, Wei Jiang, Zehuai Wen, Weihui Lu, Gaetano Marrone

**Affiliations:** ^1^Department of Global Public Health, Health Systems and Policy, Karolinska Institute, 17177 Stockholm, Sweden; ^2^Key Unit of Methodology in Clinical Research, Guangdong Provincial Hospital of Chinese Medicine, 510120 Guangzhou, China; ^3^The Second Affiliated Hospital of Guangzhou University of Chinese Medicine, 510405 Guangzhou, China; ^4^Department of Medicine, Cardiology, Karolinska Institutet, 17176 Stockholm, Sweden; ^5^Department of Cardiology, Guangdong Provincial Hospital of Chinese Medicine, 510120 Guangzhou, China; ^6^National Centre for Design Measurement and Evaluation in Clinical Research, Guangzhou University of Chinese Medicine, 510405 Guangzhou, China; ^7^Heart Failure Center/Department of Cardiology, Guangdong Provincial Hospital of Chinese Medicine, 510120 Guangzhou, China

## Abstract

**Background:**

Several randomized controlled trials (RCTs) have assessed the role of Tai Chi and Qigong Practices (TQPs) in managing chronic heart failure (CHF). They have included broad variations in comparators, sample sizes, and results. This study evaluates existing RCTs for evidence of TQPs rehabilitation effects for CHF.

**Methods:**

Both English and Chinese databases were searched from their inception to October 23, 2019. RCTs were included if they compared the addition of TQPs into routine managements (RMs) to RMs alone or compared TQPs to general exercise, with RMs as a consistent cointervention in both groups. Data were screened and extracted independently using predesigned forms. RCT quality was assessed with the Cochrane tool. The primary outcomes were peak oxygen consumption (VO_2peak_), 6-minute walking distance (6MWD), and Minnesota Living with Heart Failure Questionnaire (MLHFQ). Mean differences (MDs) and 95% confidence intervals (CIs) were calculated, and heterogeneity was assessed with an *I*^2^ statistic.

**Results:**

A total of 33 RCTs with 2,465 patients were included in the systematic review. Compared to the RMs alone, TQPs plus RMs improved VO_2peak_ (MD: 1.24 mL/kg/min, 95% CI, 0.91 to 1.57; *I*^2^ = 0%), 6MWD (MD: 59.63 meters, 95% CI, 43.35 to 75.90 *I*^2^ = 88%), and MLHFQ (MD: −8.63 scores; 95% CI, −10.60 to -6.67; *I*^2^ = 94%). Compared to general exercise, superior improvements were found in the TQP group; they were significant in MLHFQ (MD: −9.18 scores; 95% CI, −17.95 to −0.41; *I*^2^ = 86%), but not in VO_2peak_ or 6MWD. Evidence was also found of TQPs' safety and high adherence.

**Conclusions:**

Considering that there are low costs, multiple physical benefits, and no equipment required, TQPs are a promising rehabilitation therapy, as an adjunct to routine pharmacotherapies or as an alternative to conventional exercises, especially in home-based settings.

## 1. Introduction

Heart failure is a global pandemic affecting at least 26 million people worldwide, and its prevalence is increasing [1]. Although pharmacological therapy is the primary treatment, exercise-based cardiac rehabilitation (EBCR) has become an important recommendation in clinical guidelines. Unfortunately, few patients with chronic heart failure (CHF) participate in EBCR, due to the lack of resources [2]. Those patients who do participate in supervised cardiac rehabilitation programs show low adherence [3]. Therefore, finding a cheap, convenient form of EBCR with high compliance is of growing importance [4].

Qigong is an adaptable form of exercise that can be practiced at any place, any time, without any specialized equipment, and with minimal time investment. Hence, it is easily incorporated into daily routines and could be integrated into a comprehensive cardiac rehabilitation program. Qigong is an ancient Chinese martial art; it is an umbrella term, covering a spectrum of exercises including Dao-Yin-Shu (physical and breathing exercises), Wu-Qin-Xi (five animals play), Baduanjin (eight silken movements), and Yi-Jin-Jing (changing tendons exercises) [5]. Tai Chi is a well-known exercise that has grown from the Qigong tradition. Tai Chi and Qigong Practices (TQPs) typically involve slow movements synchronized with meditation and regulated breathing techniques [5]. Moreover, all TQPs share the principle that any form of Qigong has an effect on the cultivation of balance and the harmony of vital energy (qi), which functions as a holistic, coherent, and interactive system [5].

In recent years, the rehabilitating effects of TQPs for CHF patients have received increasing recognition and attention. An earlier systematic review of TQPs in cardiac rehabilitation, including seven randomized or nonrandomized controlled trials, suggested that TQPs enhance physical health and promote overall quality of life among patients with chronic heart disease [6]. This has been corroborated by a recent systematic review which included 35 randomized controlled trials (RCTs) with 2,249 patients to evaluate TQPs' effect on cardiovascular diseases [7]. However, these two reviews included studies without subgroup analysis by varying cardiovascular diseases. Therefore, the rehabilitating effects of TQPs for heart failure remain unclear.

In light of the growing number of RCTs of TQPs used for rehabilitation in CHF patients, and the ensuing need for critical evaluation, we conducted a systematic review and meta-analysis of the available evidence to inform clinical practice. The research questions were as follows: (1) Does the addition of TQPs into routine managements (RMs) improve clinical outcomes, as compared to RMs alone? (2) What is the difference between TQPs and general exercise in terms of improving CHF patients' clinical outcomes? (3) What is TQPs' safety profile and how is adherence?

## 2. Methods

This systematic review was conducted and reported in accordance with the Preferred Reporting Items for Systematic Reviews and Meta-Analyses statement (Supplementary Materials: PRISMA 2009 Checklist) [8] and the Cochrane Handbook for Interventional Reviews [9]. The study protocol has been published previously in PROSPERO (CRD42018081982).

### 2.1. Search Strategy

The electronic databases PubMed, Cochrane Central Register of Controlled Trials (CENTRAL), EMBASE, CINAHL, and China National Knowledge Infrastructure were searched from their inceptions until October 23, 2019. Searches were restricted to English and Chinese. Search strategy details are provided in the Supplemental Materials-Search strategy.

### 2.2. Study Selection

One reviewer (XC) scanned all titles and abstracts to exclude irrelevant citations which were checked by a second reviewer (YC). Two reviewers (XC and YC) independently assessed the eligibility of the remaining citations after retrieving the full texts of potentially relevant articles. Randomized controlled trials (RCTs) were included, and selection criteria conformed to the PICOS approach, as described hereinafter.

#### 2.2.1. Populations

Patients diagnosed with heart failure that were in a stable phase of the disease with no acute exacerbations were included. There were no restrictions regarding heart failure subtypes.

#### 2.2.2. Intervention

RCTs that applied an intervention group receiving any form of Qigong practice (e.g., Tai Chi, Liu-Zi-Jue, Baduanjin, Wu-Qin-Xi, and Yi-Jin-Jing) were included. However, studies were excluded if TQPs had been used in combination with other oral traditional interventions such as Chinese herbal decoctions.

#### 2.2.3. Comparators

No exercise or general exercise is planned, structured, and repetitive for the purpose of conditioning the body (such as walking, cycling, swimming, or running). RMs were provided according to clinical guidelines and as a consistent cointervention to both groups.

#### 2.2.4. Outcomes

Primary outcomes included (1) peak oxygen consumption (VO_2peak_), (2) 6-minute walking distance (6MWD), and (3) disease-specific quality of life using total scores according to the Minnesota Living with Heart Failure Questionnaire (MLHFQ). Secondary outcomes included left ventricular ejection fraction (LVEF), B-type natriuretic peptide (BNP), and other clinically relevant outcomes, as well as adverse events and participant's adherence to TQPs.

### 2.3. Data Extraction

The data extraction form was drafted based on the reporting guidelines from the extended CONSORT statement for randomized trials of nonpharmacologic treatment [10] and the TIDieR (Template for Intervention Description and Replication) [11]. One reviewer (XC) used a standardized form to extract data from the included articles. The following data were extracted: study characteristics (e.g., author, year, and country), participant characteristics such as age, sex, sample size, New York Heart Association (NYHA) functional class, LVEF subtype, TQPs interventions (e.g., TQP types, frequency, durations, and target intensity), and controls, as well as outcomes measured.

Attempts were made to contact the original investigators regarding any missing data. The extracted data was checked by a second reviewer (YC). Any discrepancies were resolved by agreement after rechecking the source papers and further discussion with a third reviewer (LM).

### 2.4. Quality and Certainty of Evidence

In accordance with recommendations in the Cochrane Handbook, the trials' methodological quality was independently evaluated by two reviewers (XC and YC) using the Cochrane risk-of-bias assessment tool. Any discrepancies were resolved by agreement after rechecking the source papers and further discussion with a third reviewer (LM). The following domains were considered: (1) random sequence generation, (2) allocation concealment, (3) blinding of patients and personnel, (4) blinding of outcome assessors for primary outcomes, (5) incomplete outcome data, and (6) selective reporting. The overall evidence and certainty of evidence were evaluated with the Grading of Recommendations Assessment, Development, and Evaluation approach.

### 2.5. Data Analysis

RevMan 5.3 (Cochrane Collaboration) and STATA 12 (StataCorp) were used to analyze the data from the included studies. The outcome measures from the individual trials were combined through meta-analysis, when possible. For each outcome measure, studies were pooled separately according to comparators: (A) TQPs plus RMs vs. RMs: to evaluate add-on effects of TQPs; and (B) TQPs plus RMs vs. general exercises plus RMs: to contrast TQPs and general exercise. For studies with multiple control groups [12, 13], such as TQPs plus RMs vs. general exercise plus RMs control vs. RMs control, the results were split into pairwise comparisons by the different comparators. For the study which had two TQP groups using the same exercise but with different practice times (30 minutes or 60 minutes) [14], the results of these two TQPs groups were combined and used as the mean effects of the TQP group.

Given that all variables in the included studies consisted of continuous data, we used the mean difference (MD) when the same instrument was used, or the standardized mean difference (SMD) when different instruments were used, with 95% confidence intervals (CIs) to analyze the outcomes. A *p* value <0.05 was considered statistically significant. For trials that had missing information for outcome means or standard deviations (SD), data was first sought from the original investigators. If it was not available from the author, then imputations were performed using the recommended statistical approaches mentioned in the Cochrane Handbook (session 6.5.2.3). For those studies using the median and interquartile range (IQR) instead of means and SD, the medians were used as a substitute for the means, and the SD was approximated as SD = IQR/1.35. For those studies with only missing SD, SD was imputed from a reported standard error, CI, *t*-statistic, *Z*-statistic, or *p* value that was correlated with the difference between the two groups' means.

Heterogeneity was assessed with a chi-square test (*p* < 0.10 was considered indicative of statistical significance) and an *I*^2^ statistic (where *I*^2^ > 30%, 50%, and 75% indicated moderate, substantial, or considerable heterogeneity, respectively) [9]. A random-effects model was employed where there was formal evidence of statistical heterogeneity (*I*^2^ statistic > 50%). Otherwise, a fixed-effects model was used. Potential publication bias was evaluated by visual examination of funnel plot asymmetry and Egger's test (*p* value <0.05 was considered statistically significant). When the number of articles included in one analysis was limited (i.e., less than 10), publication bias was not assessed.

A sensitivity analysis was conducted by removing each study individually to estimate the results' consistency. When there was heterogeneity, subgroup analyses were performed to identify its potential sources. Subgroup analyses were performed according to different types of TQPs used in the program (Tai Chi, Qigong, Tai Chi plus Qigong) and program duration (in weeks). Additionally, a post hoc subgroup analysis was conducted to explore heterogeneity originating from the heart failure subtype (HFrEF or HFpEF).

## 3. Results

In total, 1,480 records were retrieved from database searches. After excluding duplicates, 999 potentially relevant abstracts were screened, and 934 were excluded for failing to meet the inclusion criteria. The remaining 65 full texts were read, and 33 RCTs [12–44] (24 in Chinese [12, 14, 18–22, 24–30, 34–42, 44] and 9 in English [13, 15–17, 23, 31–33, 43]) were deemed eligible for this systematic review. The quantitative synthesis was performed with 32 RCTs by pooling the results through a meta-analysis. A flowchart of the study selection process is shown in eFigure 1 in Supplementary Materials.

### 3.1. Characteristics of Included Studies

#### 3.1.1. Study Characteristics

The characteristics of the included studies are shown in Table 1. The studies were published between 2004 and 2019. States or regions of publication were China (*n* = 25) [12, 14, 17–22, 24–30, 34–42, 44], Taiwan (*n* = 2) [17, 44], the United States (*n* = 5) [13, 23, 31–33], the United Kingdom (*n* = 1) [15], Italy (*n* = 1) [16], and Sweden (*n* = 1) [43].

#### 3.1.2. Participants

The included RCTs involved a total of 2,465 CHF patients (age ranging from 52 to 74 yrs) with NYHA functional class ranging from I to IV. The sample size per RCT ranged from 16 to 180, 84% of whom were Chinese.

#### 3.1.3. Intervention

For the TQP exercise program type, researchers used Tai Chi in 17 RCTs [13, 14, 16, 18, 19, 22–25, 30–33, 35, 38, 41, 43], Qigong in 14 RCTs, and Tai Chi plus Qigong in the remaining two RCTs [15, 40]. Qigong included Baduanjin [17, 21, 26, 27, 36, 37, 39, 42], Liuzijue [12], Baduanjin plus Liuziju [20, 28, 29, 34], and Chan-Chuang [44]. TQP exercise time lasted from 15 to 60 minutes per session, and TQP program duration varied between 4 weeks (*n* = 2) [19, 36], 8 weeks (*n* = 1) [21], 12 weeks (*n* = 17) [12, 16, 17, 23–27, 31–33, 35, 37–40, 44], 16 weeks (*n* = 3) [13, 15, 43], 24 weeks (*n* = 5) [14, 18, 22, 30, 41], and 52 weeks (*n* = 5) [20, 28, 29, 34, 42].

#### 3.1.4. Comparators

In terms of the comparison, 26 studies compared TQPs plus RMs against RMs alone [15–20, 22–24, 26–32, 34–36, 38–44]. Five studies compared TQPs plus RMs against general exercises plus RMs [14, 21, 25, 33, 37], one of which applied two TQP groups using Tai Chi with different practice times (30 minutes vs. 60 minutes) [14]. There were two 3-arm studies that included two control groups comparing TQPs plus RMs vs. general exercises plus RMs vs. RMs [12, 13]. Generally, RMs included standard pharmacological treatments in all of the RCTs; in some of the RCTs, it also included education, dietary counseling, and/or general exercise advice.

#### 3.1.5. Outcomes

**Figure 1:**
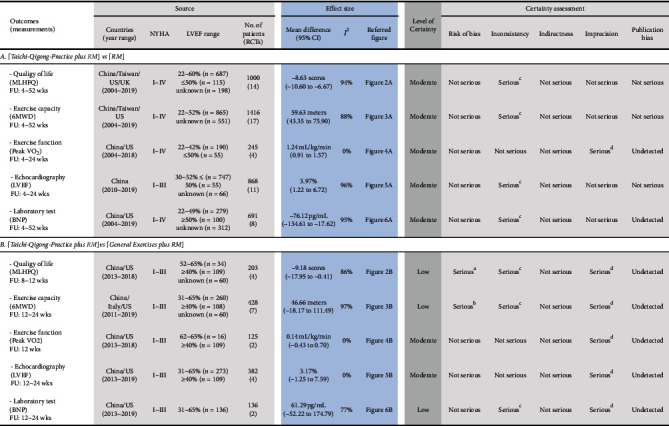
Summary of findings. a: 30% of the information from 1 high-risk RCT; removing this RCT significantly altered the effect estimates; 25% of the studies had a high risk of the randomization process. b: 20% of the information from 1 high-risk RCT; removing this RCT moderately altered the size of effect estimates; 17% of the studies had a high risk of the randomization process. c: considerable heterogeneity based on *I*^2^. d: imprecision, as OIS criteria were not met, primarily due to the small sample size (<400). 6MWD: 6-minute walking distance, BNP: B-type natriuretic peptide, CI: confidence interval, FU: follow-up, LVEF: left ventricular ejection fraction, MLHFQ: Minnesota Living with Heart Failure Questionnaire, NYHA: New York Heart Association, Peak VO2: peak oxygen consumption, RCT: randomized controlled trial, RM: routine management (according to current guidelines).

Results of three primary outcomes (MLHFQ, 6MWD, and VO_2peak_) and two secondary outcomes (LVEF and BNP) are summarized in Figure 1.

### 3.2. MLHFQ

**Figure 2:**
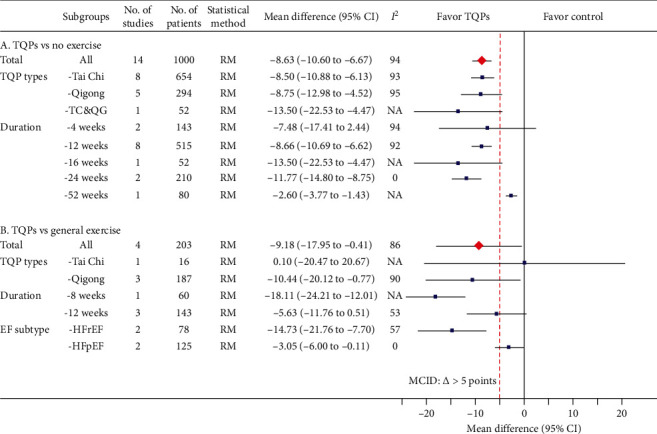
Meta-analysis results for MLHFQ (total score) including overall pooled effects and subgroup effects. The pooled effect from all included studies and each subgroup is shown in red diamond and blue square, respectively. Both are along with their 95% confidence intervals. Meta-analysis results showing individual study data are presented in Supplementary Materials: eFigures 2 and 3 for part A and part B, respectively. MLHFQ: Minnesota Living with Heart Failure Questionnaire, No.: number, CI: confidence interval, TQP: Tai Chi and Qigong Practice, NA: not available, TC&QG: Tai Chi and Qigong, RM: random-effects model, MCID: minimum clinically important difference, EF: ejection fraction, HFrEF: heart failure with reduced ejection fraction, HFpEF: heart failure with perceived ejection fraction.

(A) *TQPs plus RMs versus RMs.* The addition of TQPs produced a statistically significant lower MLHFQ score (better quality of life), but the heterogeneity was considerable (14 RCT [12, 15, 17–19, 24, 26, 30–32, 35, 36, 38, 42], *n* = 10,000; MD: −8.63 scores; 95% CI, −10.60 to −6.67; *I*^2^ = 94%; Figure 2A). The subgroup analysis based on TQP types and program durations did not resolve the heterogeneity (Figure 2A).

(B) *TQPs versus General Exercise*. The pooled results also showed statistically significant lower MLHFQ scores in the TQPs group than in the general exercise group, but again heterogeneity was considerable (4 RCTs [12, 21, 33, 37], *n* = 203; MD: −9.18 scores; 95% CI, −17.95 to −0.41; *I*^2^ = 86%; Figure 2B). Subgroup analyses revealed that TQP durations and HF subtype might explain the heterogeneity (Figure 2B).

### 3.3. 6MWD

#### 3.3.1. TQPs plus RMs versus RMs

**Figure 3:**
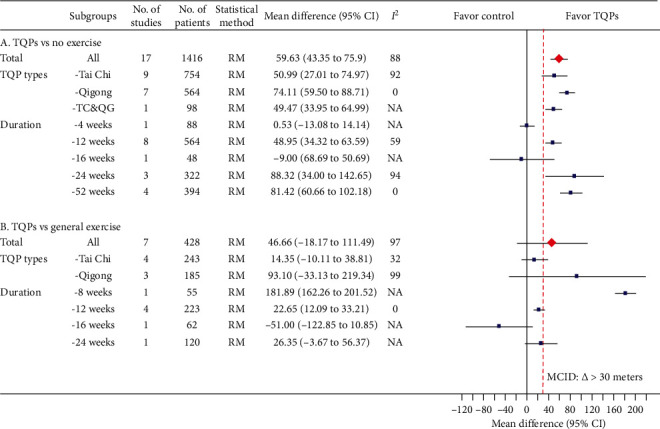
Meta-analysis results for 6MWD (meters) including overall pooled effects and subgroup effects. The pooled effect from all included studies and each subgroup is shown in red diamond and blue square, respectively. Both are along with their 95% confidence intervals. Meta-analysis results showing that individual study data are presented in Supplementary Materials: eFigures 4 and 5 for part A and part B, respectively. 6MWD: 6-minute walking distance, No.: number, CI: confidence interval, TQP: Tai Chi and Qigong Practice, NA: not available, TC&QG: Tai Chi and Qigong, RM: random-effects model, MCID: minimum clinically important difference.

The pooled results from 17 RCTs [12, 13, 19, 20, 22, 24, 26, 28–32, 34, 38, 40, 41, 44] (*n* = 1,416) showed that adding TQPs led to a statistically significant 56.52 meter increase in 6MWD (95% CI, 41.27 to 71.78), but the heterogeneity was considerable (*I*^2^ = 88%; Figure 3A). In the subgroup analyses, the heterogeneity declined in the Qigong subgroup and the 12-week subgroup (Figure 3A).

#### 3.3.2. TQPs versus General Exercise

The pooled results, though not statistically significant, showed that TQPs were more effective, but with considerable heterogeneity (7 RCTs [12–14, 16, 21, 33, 37], *n* = 428; MD: 46.66 meters; 95% CI, −18.17 to 111.49; *I*^2^ = 98%) (Figure 3B). Deleting the study with the stronger effect [21] reduced the heterogeneity to *I*^2^ = 20%, without changing the results' significance (Supplementary Materials: eTable 1**)**. TQP durations might also explain the heterogeneity (Figure 3B).

### 3.4. VO_2peak_

#### 3.4.1. TQPs plus RMs versus RMs

**Figure 4:**
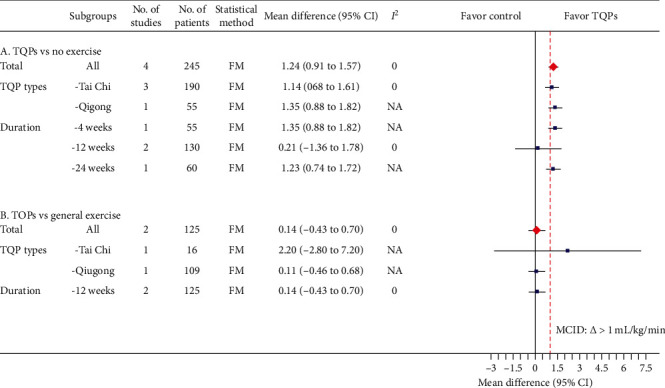
Meta-analysis results for peak VO2 (mL/kg/min) including overall pooled effects and subgroup effects. The pooled effect from all included studies and each subgroup is shown in red diamond and blue square, respectively. Both are along with their 95% confidence intervals. Meta-analysis results showing that individual study data are presented in Supplementary Materials: eFigures 6 and 7 for part A and part B, respectively. VO2: oxygen consumption, No.: number, CI: confidence interval, TQP: Tai Chi and Qigong Practice, NA: not available, RM: random-effects model, FM: fixed-effects model, MCID: minimum clinically important difference.

The pooled results from 4 RCTs [18, 31, 32, 36, 37] (*n* = 245) showed that adding TQPs into RMs can lead to a statistically significant 1.24 mL/kg/min improvement in patients' VO_2peak_ (95% CI, 0.91 to 1.57; *I*^2^ = 0%; Figure 4A).

#### 3.4.2. TQPs versus General Exercise

The pooled MD, although not statistically significant, showed that TQPs were more effective (2 RCTs [33, 37], *n* = 125; MD: 0.14 mL/kg/min, 95% CI, −0.43 to 0.7; *I*^2^ = 0%; Figure 4B). Both of these RCTs included HFpEF patients, and both lasted for 12 weeks.

### 3.5. Secondary Outcomes

**Figure 5:**
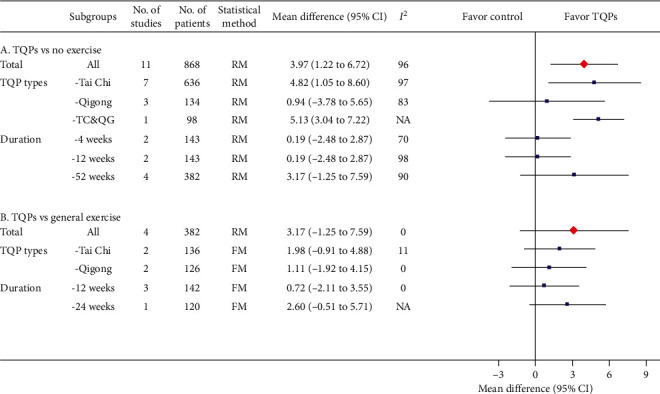
Meta-analysis results for LVEF (%) including overall pooled effects and subgroup effects. The pooled effect from all included studies and each subgroup is shown in red diamond and blue square, respectively. Both are along with their 95% confidence intervals. Meta-analysis results showing that individual study data are presented in Supplementary Materials: eFigures 8 and 9 for part A and part B, respectively. LVEF: left ventricular ejection fraction, No.: number, CI: confidence interval, TQP: Tai Chi and Qigong Practice, NA: not available, TC&QG: Tai Chi and Qigong, RM: random-effects model, FM: fixed-effects model, MCID: minimum clinically important difference.

**Figure 6:**
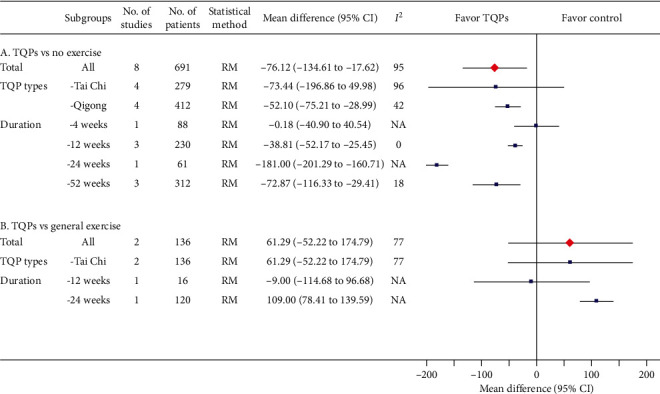
Meta-analysis results for BNP (pg/mL) including overall pooled effects and subgroup effects. The pooled effect from all included studies and each subgroup is shown in red diamond and blue square, respectively. Both are along with their 95% confidence intervals. Meta-analysis results showing that individual study data are presented in Supplementary Materials: eFigures 10 and 11 for part A and part B, respectively. BNP: B-type natriuretic peptide, No.: number, CI: confidence interval, TQP: Tai Chi and Qigong Practice, NA: not available, RM: random-effects model, MCID: minimum clinically important difference.

The addition of TQPs showed a small but significant improvement in the LVEF (Figure 5A), as well as in the BNP (Figure 6A). Similarly, when compared to general exercises, the pooled MD showed that TQPs were more effective for the LVEF (Figure 5B), as well as for the BNP (Figure 6B). However, neither was statistically significant. Results of other secondary clinical outcomes reported in more than one study are presented in Table 2.

### 3.6. Sensitivity Analysis

Sensitivity analysis showed that most of the pooled results were robust when removing each study individually. When comparing TQPs with general exercises, the beneficial effects of TQPs on MLHFQ became statistically insignificant if 2 studies were removed individually (Supplementary Materials: eTable 1). For the pool of 6MWD comparing TQPs with general exercises, deleting one study [21] made the beneficial effects of TQPs significant and reduced the heterogeneity from *I*^2^ = 97% to *I*^2^ = 20% (Supplementary Materials: eTable 1**)**. In the BNP sensitivity analysis, deleting Pan's study [22] reduced the heterogeneity from *I*^2^ = 95% to *I*^2^ = 39%, without changing the results' significance (Supplementary Materials: eTable 1**)**.

### 3.7. TQP Safety and Patient Adherence

No adverse event related to TQPs was found in the included studies, and patient dropout in the TQP groups was low, with most withdrawals being due to hospitalization or CHF exacerbation. TQP adherence was good. The retention rate in the TQP groups ranged from 67% to approximately 100%. The six studies that included TQP training classes reported attendance between 75% and 89% [13, 23, 31–33, 43] (Table 1).

### 3.8. Evidence Quality and Certainty

Individual items on the risk-of-bias assessment are shown in Figures 12 and 13 in Supplementary Materials. As TQP is an exercise training, designing an experiment with a credible placebo-control arm is challenging. Thus, all RCTs were open-label. All studies were unclear on outcome assessment blinding except for two RCTs [32, 33] in which the authors claimed that the outcome assessors had been blind to patient treatment allocation. Only half of the RCTs provided adequate random sequence generation [16–19, 22, 25, 29, 35–40, 44], and only five RCTs reported allocation concealment methods [13, 17, 31, 37, 44]. Twenty-nine (88%) studies were at unclear risk of selective reporting because neither protocol nor trial registration info was available.

Publication bias analyzed by funnel plots showed only minor asymmetry (Supplementary Materials: eFigure 14), and there was no evidence of funnel plot asymmetry for 6MWD (*p*=0.10), MLHFQ (*p*=0.817), or LVEF (*p*=0.929). Thus, a publication bias mechanism is not a major cause for concern.

Evidence from RCTs indicated (with a moderate level of certainty) that the addition of TQPs into RMs was associated with a better quality of life, improved exercise capacity, increased LVEF, and reduced BNP level, as compared with the associated with the RMs alone. Low evidence certainty showed that TQPs were associated with a larger improvement in the quality of life and exercise capacity than general exercise (Figure 1).

## 4. Discussion

This systematic review identified 33 RCTs with a total of 2,465 participants to evaluate the evidence of rehabilitative effects from TQPs for patients with CHF. There are three main findings. (1) When compared to RMs, TQPs plus RMs improved VO_2peak_, 6MWD, and MLHFQ, and the pooled effects were robust with heterogeneity found in 6MWD and MLHFQ, but not in VO_2peak_. In addition, TQPs might have beneficial effects on other clinical outcomes such as LVEF and BNP. (2) When compared to general exercise, superior improvements were found in the TQP group; they were significant in MLHFQ, but not in VO_2peak_, 6MWD, LVEF, or BNP. (3) Evidence was also found that TQP is safe and that there is high adherence to TQP programs.

Quality of life and exercise capacity are two different domains of interest in rehabilitation research [45]. The positive results of MLHFQ suggest that TQPs can improve CHF patients' quality of life. The present findings are consistent with those of other systematic reviews and meta-analyses of RCTs of Tai Chi for CHF [46–48]. Moreover, the pooled effects are also clinically significant, with the effect estimate being greater than the minimally important clinical difference of 5 points [49] (Figure 2). TQPs are characterized by the interplay between flowing circular physical postures and movements, mindful awareness, and breathing techniques in a harmonious manner [5]. Hence, they exert less cardiopulmonary stress and enable the body to relax after practicing. They also address breathing, a crucial aspect of CHF management. Slowing breathing patterns allows more complete inspiration/expiration and gas exchange in patients with CHF [50]. This mitigates their symptoms, thus improving their quality of life.

We found a moderate level of evidence that the addition of TQPs into RMs benefits CHF patients' exercise capacity. Firstly, positive results were found for the 6MWD, both in the overall pooling of 17 studies (60 meters) and in each of the TQP subgroups. Our results are consistent with a previous systematic review of Tai Chi which reported a similar improvement of 50 meters [46]. The pooled improvements were also clinically relevant because they are larger than the minimum clinically important difference (>30 meters) [51].

Secondly, positive results were also found for the VO_2peak_ which is the gold standard for assessing exercise capacity [52]. This reinforces the beneficial gains in exercise capacity by practicing TQPs for CHF patients. Unlike findings from previous reviews which reported that Tai Chi could not change the VO_2peak_ [46–48], there was a positive result for VO_2peak_ in the Tai Chi subgroup in our study. Our meta-analysis involves two more recent RCTs [18, 36] which were not included in those previous meta-analyses. This results in a larger sample size. In addition, we have restricted our attention to those studies without exercise controls. Studies with exercise controls, similar to the active-control in the pharmacological trials, are expected to have negative results and the effects' estimates are usually smaller than those of placebo-control trials, especially for the open-label studies.

Evidence from the RCTs indicated (with a low level of certainty) that TQPs may have rehabilitation effects on quality of life and exercise capacity that are similar to those of general aerobic exercises. Our findings show that TQPs' benefits are superior to those of general exercise at improving quality of life (MD: −9.18). This has not been reported in earlier studies. The effect size is similar to that reported in a Cochrane systematic review [53], in which the reviewers compared all exercise interventions with usual care (−7.11 points, 95% CI −10.49 to −3.73). In addition, the magnitude of the pooled improvements from the TQPs in VO_2peak_ and 6MWD was similar to that of the pooled improvements from conventional exercise modalities in other systematic reviews, respectively [54, 55]. Moreover, although the pooled effect estimate for 6MWD was not statistically significant when compared to general exercise controls, it is clinically significant as it exceeds the minimum important clinical difference of 30 meters [51].

The improvements in LVEF and BNP provide further objective evidence (with a moderate level of certainty) for TQPs' benefit in patients with CHF. The pooled effect of LVEF and BNP is consistent with previous research [46]. The possible mechanisms for the improvement observed in LVEF and BNP in trials of TQP therapy have yet to be established. TQPs, as a moderate-intensity exercise, could improve the parasympathetic nervous degree, inhibit sympathetic activity, and increase coronary collateral circulation, cardiac stroke volume, and cardiac output. In this way, it could reduce BNP and increase LVEF [56].

It is also worth noting that no adverse events were reported, and patient dropout in the TQP groups was low, with most withdrawals being due to hospitalization or CHF exacerbation. The included RCTs showed that participants had good adherence to TQP programs. These results indicate that TQPs are safe and engender good patient compliance.

## 5. Limitations

There are several limitations to this review:

  Firstly, the interpretation of our results may be challenged by the heterogeneity observed. Sensitivity analyses by TQP styles or durations revealed some sources of heterogeneity but were unable to account for all of the variations. For example, practitioner expertise, heart failure etiopathogenesis, NYHA classification, and patients with different cultural backgrounds were each revealed to be potential sources of heterogeneity. As many individuals or combined factors may have influenced heterogeneity, this study did not succeed in identifying the reasons for this.
  Secondly, the descriptions of the 33 RCTs regarding the randomization method, allocation concealment, and blinding evaluation were neither detailed nor comprehensive. Therefore, the included studies might exhibit moderate selection, implementation, and measurement biases. Because of the intervention itself, participants could identify they were in the experimental group. Trials with inadequate blinding are likely to exaggerate treatment effects, especially with regard to subjective results and with participants with knowledge of Chinese traditional culture.
  Thirdly, training characteristics regarding training intensity are rarely described adequately enough to evaluate TQP's dose-response effects, i.e., to render the studies replicable, or to interpret the findings' validity and translate the interventions into practice. This might be due to the fact that there is still no standardized method to measure TQPs' intensity. As exercise intensity is reported to be the most critical component for improving cardiorespiratory fitness [57], future research on TQPs should provide descriptions of intensity, in combination with frequency, duration, and adherence.
  Lastly, the majority of this review's sample is from China, but people from different cultures may experience variegated responses to TQPs. Moreover, rehabilitation-related trials have been investigating the efficacy of exercises in selective populations, such as women, the elderly, ethnic minorities, HFpEF, and high-risk patients. Future research might also identify subgroups that benefit the most/least from TQPs.

## 6. Conclusions

The findings of this systematic review and meta-analysis suggest that based on moderate-level evidence, adding TQPs into RMs was associated with statistically significant improvements to the quality of life and exercise capacity in CHF patients. In addition, evidence from RCTs indicated (with a low level of certainty) that TQPs may have similar rehabilitation effects as general aerobic exercises. Considering the lack of special equipment requirements, low costs, and multiple physical benefits, TQPs may represent a promising rehabilitation therapy as an adjunct to routine pharmacotherapies or as an alternative to conventional exercises. Incorporation into cardiac rehabilitation programs for patients with CHF should be considered, especially in home-based settings.

## Figures and Tables

**Table 1:** Characteristics of included studies.

Source (country)	Populations				TQPs (time/frequency]	Period (weeks)	Comparison (A^a^/B^b^)	Routine management^c^	Outcomes	Adherence to TQPs
	NYHA class	Sample size (drop out) (I/C), #	Male (I/C), %	Age, yrs (I/C) mean ± SD						
Barrow et al. [15] (UK)	II∼III	65 (32/33) (13 (7/6))	81%/82%	68.4 ± NA^d^/67.9 ± NA^d^	III. Tai Chi and Qigong (55 mins/twice per week)	16	A^a^	TDs^e^	MLHFQ, SCL-90-r (depression and anxiety), SBP DBP, ISWT	78% retention rate; practice for >1 h/week: *n* = 14; <1 h/week: *n* = 11
Caminiti et al. [16] (Italy)	II	60 (30/30) (0)	83%/87%	74.1 ± 6.0/73.4 ± 2.0	I. Tai Chi (30 min/twice per week)	12	B^b^ (cycling: 30 mins/twice per week)	TDs^e^; walking: (30 min/twice per week)	6MWD, SBP, DBP, NTpro-BNP, strength, heart rate, QoL-VAS, MacNewQL	100% retention rate
Chen et al. [17] (Taiwan)	I∼II	80 (39/41) (17 (9/8))	46%/59%	70.3 ± 13.5	II. Qigong (*Baduanjin*) (35 min/3 times per week)	12	A^a^	TDs^e^	MLHFQ, fatigue (PFS)	77% retention rate
Feng [18] (China)	II-III	60 (32/28) (0)	50%/54%	60.6 ± 6.5/61.6 ± 5.1	I. Tai Chi (30 min/3∼5 times per week)	24	A^a^	TDs^e^; dietary advice	VO_2peak_, MLHFQ, VO_2AT_, O_2_ pulse, NT-proBNP, LVEF	100% retention rate
Huang [19] (China)	III	88 (44/44) (0)	48%/50%	64.3 ± 6.5/64.5 ± 5.9	I. Tai chi (30 min/daily)	4	A^a^	TDs^e^; walking (30 min/daily)	6MWD, MLHFQ, LVEF, LVEDd, BNP	100% retention rate
Li et al. [20] (China)	II∼IV	180 (90/90) (0)	57%	70.5 ± 4.5	II. Qigong (*Baduanjin and Liu-Zi-Jue*) (NA^d^)	52	A^a^	TDs^e^; supervision; daily life advice	6MWD, BNP	100% retention rate
Li [21] (China)	II∼III	60 (30/30) (0)	60%/67%	69.2 ± 6.6/66.6 ± 5.3	II. Qigong (*Baduanjin*) (4∼8 sets/daily)	8	B^b^ (walking: 1,000 meters/twice per day)	TDs^e^	6MWD, MLHFQ	100% retention rate
Pan [22] (China)	II-III	61 (31/30) (0)	58%/57%	67.7 ± 12.1/66.2 ± 11.8	I. Tai Chi (≥30 min/daily)	24	A^a^	TDs^e^; dietary advice	6MWD, LVEF, LVEDd, BNP, cTNT, SF-36	100% retention rate
Redwine et al. [23] (US)	II	24 (12/12) (4 (4/0))	83%/92%	72.6 ± 6.2/63.9 ± 12.0	I. Tai Chi (60 min/twice per week)	12	A^a^	TDs^e^; regular medical visits	BDI (depression), MFSI-S (fatigue)	67% retention rate; 87.5% class attendance rate
Sang [24] (China)	II-III	100 (50/50) (0)	56%/58%	65.3 ± 8.2/76.2 ± 7.5	I. Tai Chi (15 min/daily)	12	A^a^	TDs^e^	6MWD, MLHFQ, LVEF, AngII, BNP,	100% retention rate
Wang [25] (China)	I∼III	56 (28/28) (0)	43%/36%	67.0 ± 1.8/66.7 ± 2.0	I. Tai Chi (30 min/5 times per week)	12	B^b^ (walking, 30 min/5 times per week)	TDs^e^	6MWD, SF-36	100% retention rate
Xiong and Xu [26] (China)	II∼III	63 (33/30) (0)	61%/60%	70.3 ± 6.4/69.7 ± 7.2	II. Qigong (*Baduanjin*) (30 min/daily)	12	A^a^	TDs^e^	6MWD, MLHFQ, LVEF, NT-proBNP	100% retention rate
Xiong [27] (China)	II∼III	60 (30/30) (0)	63%/60%	70.1 ± 6.3/69.4 ± 7.1	II. Qigong (*Baduanjin*) (30 min/daily)	12	A^a^	TDs^e^	MMSE (mental state), ADL	100% retention rate
Yan [28] (China)	II∼IV	72 (36/36) (0)	56%/61%	71.4 ± 6.5/73.4 ± 6.9	II. Qigong (*Baduanjin and Liu-Zi-Jue*) (NA^d^)	52	A^a^	TDs^e^; supervision; daily life advice	6MWD, BNP	100% retention rate
Yang [29] (China)	II∼IV	60 (30/30) (0)	53%/50%	70.5 ± 4.9/68.5 ± 4.9	II. Qigong (*Baduanjin and Liu-Zi-Jue*) (15–30 min/5 times per week)	52	A^a^	TDs^e^; supervision; daily life advice	6MWD, BNP	100% retention rate
Yao et al. [30] (China)	II	150 (80/70) (0)	59%/60%	52.4 ± 6.3/51.7 ± 7.3	I. Tai Chi (≥30 mins/5 times per week)	24	A^a^	TDs^e^; daily life advice	6MWD, MLHFQ, LVEF, LVEDd	100% retention rate
Yeh et al. [31] (US)	I∼IV	30 (15/15) (0)	67%/60%	66.0 ± 12.0/61.0 ± 14.0	I. Tai Chi (60 min/twice per week)	12	A^a^	TDs^e^; dietary counsel; exercise advice	6MWD, VO_2peak,_ MLHFQ, BNP, norepinephrine	100% retention rate, 83% class attendance rate, 93% reported home practice for 86 min/week
Yeh et al. [32] (US)	I∼III	100 (50/50) (4 (1/3))	56%/72%	68.1 ± 11.9/66.6 ± 12.1	I. Tai Chi (60 min/twice per week)	12	A^a^ (education)	TDs^e^; dietary counsel; exercise advice	6MWD, VO_2peak_, MLHFQ, Norepinephrine, CRP, TNF, Endothelin, TUG, BNP, POMS, SEE	92% retention rate, 75% class attended rate, 93% home practice for 48 min/week
Yeh et al. [33] (US)	I∼III	16 (8/8) (0)	50%/50%	68.0 ± 11.0/63.0 ± 11.0	I. Tai Chi (60 min/twice per week)	12	B^b^ (aerobic exercise: 60 min/twice per week)	TDs^e^; dietary counsel; exercise advice	6MWD, VO_2peak_, MLHFQ, TUG, BNP, POMS, SEE, SBP, DBP, LVEF, LAD, LAV, E/A	100% retention rate, 89% class attendance rate
Yu [34] (China)	II∼IV	82 (41/41) (0)	46%/49%	72.1 ± 8.4/71.5 ± 8.4	II. Qigong (*Baduanjin and Liu-Zi-Jue*)(15∼30 min/daily)	52	A^a^	TDs^e^; daily life advice	6MWD	100% retention rate
Yuan [35] (China)	II∼III	60 (30/30) (0)	57%/53%	66.3 ± 5.6/67.5 ± 3.8	I. Tai Chi (20–40 min/5 times per week)	12	A^a^	TDs^e^; education; antidepressants	MLHFQ, HAMD (depression), PSQI (sleep)	100% retention rate
Zheng et al. [12] (China)	II∼III	28 (11/9/8) (4(2/1/1))	67%/71%/75%	59.5 ± 7.2/59.1 ± 9.1_C1_/58.9 ± 8.6_C2_	II. Qigong (*Liu-Zi-Jjue*) (30 min/daily)	12	A^a^_C1_; B^b^_C2_(walking, 30 min/daily)	TDs^e^	6MWD, MLHFQ, LVEF, NT-proBNP	82% retention rate; averaged practice time: 19.7 ± 9.9 mins per day
Shi [36] (China)	II∼III	55 (26/29) (5(4/1))	46%/59%	58.6 ± 4.2/58.2 ± 4.4	II. Qigong (*Baduanjin*) (30 min/daily)	4	A^a^	TDs^e^	VO_2peak_, MLHFQ, VO_2AT_, VE/VCO_2_, NT-proBNP, LVEF	85% retention rate
Yu [37] (China)	I∼II	109 (54/55) (11 (6/5))	42%/39%	60.3 ± 8.8/60.7 ± 9.7	II. Qigong (*Baduanjin*) (45 min/2–3 times per week)	12	B^b^ (rehab exercises: 45 min/twice weekly)	TDs^e^; education; exercise advice	6MWD, VO_2peak_, MLHFQ, VO_2AT_, METs, LVEF, LVEDd, NT-proBNP, CRP	89% retention rate
Liu [38] (China)	II∼III	66 (33/33) (0)	45%/48%	55.2 ± 1.3/54.8 ± 1.3	I. Tai Chi (30–60 min/3-4 times per week)	12	A^a^	TDs^e^	6MWD, MLHFQ, LVEF	100% retention rate
Li [39] (China)	II∼III	100 (50/50) (0)	74%/70%	65.3 ± 7.9/62.0 ± 9.3	II. Qigong (*Baduanjin*) (20∼30 min/≥5 times per week)	12	A^a^	TDs^e^	MLHFQ, BNP, CgA	100% retention rate
Yu [14] (China)	I∼III	120 (40/40/40) (0)	23%/24%/26%	58.8 ± 11.2_I1_/59.4 ± 12.1_I2_/61.8 ± 12.7^C^	I. Tai Chi (30 min^I1^, 60 min^I2^/5 times per week)	24	B^b^ (walking, 30 min/once daily)	TDs^e^	6MWD, BNP, LVEF	100% retention rate
Yu and Jiang [40] (China)	II∼III	98(49/49) (0)	65%/61%	65.5 ± 7.0/65.8 ± 7.1	III. Tai Chi and Qigong (*Baduanjin*) (30 min/once daily^Tai Chi^; 30 min 5 times weekly^BDJ^)	12	A^a^	TDs^e^	6MWD, VO_2peak_, LVESd, LVEDd, LVEF, E/A, VO_2_/HR, SF-36	100% retention rate
Deng et al. [41] (China)	I∼III	113 (57/56) (2 (2/0))	54%/52%	64.7 ± 4.2/67.2 ± 4.9	I. Tai Chi (40∼60 min/≥5 times per week)	24	A^a^	TDs^e^; daily life advice	6MWD, NT-proBNP, LVEF, HAMA (anxiety), HAMD (depression)	96% retention rate
Lu [42] (China)	III-IV	80 (40/40) (0)	80%/23%	69.0 ± 6.8/68.6 ± 7.6	II. Qigong (*Baduanjin*) (30 min/twice per day)	52	A^a^	TDs^e^	MLHFQ	100% retention rate
Hagglund et al. [43] (Sweden)	NA^d^	45 (25/20) (11 (5/6))	76%/80%	76(71–85)/76(71–83)	I. Tai Chi (60 min/twice per week)	16	A^a^	TDs^e^	MLHFQ, NT-proBNP, MFI-20 (fatigue), SPPB (balance),	96% retention rate; 72% of participants completed ≥75% of sessions
Redwine et al. [13] (US)	NA^d^	70 (25/23/22) (7 (4/0/3))	92%/86%/87%	63.0 ± 9.0/67.0 ± 7.0_C1_/65.0 ± 9.0_C2_	I. Tai chi (60 min/twice per week)	16	A^a^_C1_; B^b^_C2_ (resistant band train: 60 min/twice per week)	TDs^e^; usual care	6MWD, BDI (depression), LVESd, LVEF	84% retention rate; 87% class attendance rate; a median of 74 min/week practice time
Zheng [44] (Taiwan)	II	91(41/44) (9 (3/6))	72%/70%	62.2 ± 15.1/66.6 ± 12.7	II. Qigong (*Chan-Chuang*) (≥15 min/2∼3 times per day)	12	A^a^	TDs^e^	6MWD, HADS (depression), SF-12	93% retention rate

a. A: TQPs versus no TQPs or other exercises; b. B: TQPs versus general exercises; c. Routine managements were given to all participants; d. Data are not available from original papers; e. Therapeutic drugs were prescribed according to heart failure management guidelines. NYHY: New York Heart Association, I: intervention group, C: control group, SD: standard deviation, TQPs: Tai Chi-Qigong Practices, NA: not available, TDs: therapeutic drugs, MLHFQ: Minnesota Living with Heart Failure Questionnaire, SCL-90-R: symptom checklist-90-revised, SBP: systolic blood pressure, DBP: diastolic blood pressure, ISWT: incremental shuttle walk test, 6MWD: 6-minute walking distance, NTpro-BNP: N-terminal B-type natriuretic peptide, QoL-VAS: quality of life assessment by a visual analog scale, MacNewQL: MacNew quality of life after myocardial infarction questionnaire, PFS: piper fatigue scale, VO_2peak_: peak oxygen consumption, VO_2AT_, oxygen consumption at anaerobic threshold, O_2:_ oxygen, LVEF: left ventricular ejection fraction, LVEDd: left ventricular end-diastolic diameter, BNP: B-type natriuretic peptide, cTNT: cardiac troponin-T, BDI: beck depression inventory, MFSI-S: multidimensional fatigue symptom inventory–short form, AngII: angiotensin II, MMSE: minimental state examination, ADL: daily activities, CRP: C-reactive protein, TNF: tumor necrosis factor, TUG: Time Up and Go test, POMS: profile of mood state, SEE: self-efficacy for exercise, LAD: left atrial dimension, LAV: left atrial volume, HAMD: Hamilton depression rating scale, PSQI: Pittsburgh sleep quality index, VE/VCO_2_: ventilatory equivalents for carbon dioxide, CgA: chromogranin A, LVESd: left ventricular end-systolic diameter, HAMA: Hamilton anxiety rating scale, MFI-20: multidimensional fatigue inventory-20 items, SPPB: short physical performance battery, HADS: hospital anxiety and depression scale.

**Table 2:** Meta-analysis results of clinical outcomes with more than one study.

Outcomes measurements	No. of RCTs	No. of patients	Statistical method	Effect sizes		Heterogeneity (*I*^2^) (%)
				MD (95% CI)	*p* value	
*A. (Tai Chi and Qigong Practice plus RM) vs. (RM)*						
NT-proBNP, pg/mL	6	350	MD, REM	−232.05 (−578.87 to 114.78)	0.19	97
VO_2AT_, ml/kg/min	2	115	MD, REM	1.43 (0.59 to 2.28)	0.0009	93
LVEDd, mm	4	397	MD, REM	−3.00 (−5.09 to −0.91)	0.005	63
Depression	6	462	SMD, REM	−0.64 (−1.03 to −0.25)	0.001	76
Anxiety	2	163	SMD, FEM	−1.00 (−2.41 to 0.41)	0.17	94
Mood state	2	160	SMD, REM	−0.08 (−1.47 to 1.31)	0.91	94
Fatigue	2	125	SMD, REM	0.01 (−1.07 to 1.09)	0.98	88
Norepinephrine	2	130	MD, FEM	0.51 (−0.71 to 1.72)	0.41	0
SF-36-bodily pain	2	159	MD, REM	5.84 (0.62 to 11.06)	0.03	80
SF-36-mental health	2	159	MD, REM	6.55 (1.78 to 11.32)	0.007	58
SF-36-physical function	2	159	MD, FEM	6.73 (4.05 to 9.42)	<0.00001	20
SF-36-role emotional	2	159	MD, FEM	5.60 (2.78 to 8.43)	<0.0001	48
SF-36-role physical	2	159	MD, REM	9.87 (0.52 to 19.22)	0.04	92
SF-36-social function	2	159	MD, FEM	6.78 (4.09 to 9.47)	<0.00001	0
SF-36-vitality	2	159	MD, REM	8.28 (0.77 to 15.79)	0.03	91
Hospitalizations per capita	3	322	MD, FEM	−0.82 (−0.95 to −0.69)	<0.00001	*I* ^2^ = 0
Hospitalization cost per capita	3	322	MD, FEM	−1.60 (−1.85 to −1.36)	<0.00001	*I* ^2^ = 0

*B.(Tai Chi and Qigong Practice plus RM) vs. (general exercises plus RM)*						
NT-proBNP, pg/mL	3	186	MD, FEM	−12.01 (−23.65 to −0.37)	0.04	0
SBP, mmHg	2	76	MD, FEM	−9.60 (−22.02 to 2.83)	0.13	0
DBP, mmHg	2	76	MD, FEM	0.82 (−5.27 to 6.92)	0.79	0
Depression	2	63	SMD, FEM	−0.28 (−0.77 to 0.22)	0.28	0

RCT: randomized controlled trial, CI: confidence interval, RM: routine management, NT-proBNP: *N*-terminal B-type natriuretic peptide, VO_2AT_: oxygen consumption at anaerobic threshold, MD: mean difference, SMD: standardized mean difference, REM: random-effects model, FEM: fixed-effects model, LVEDd: left ventricular end-diastolic end-systolic diameters, SBP: systolic blood pressure, DBP: diastolic blood pressure.

## Data Availability

Data are available upon request to the corresponding author.

## Abbreviations

6MWD: 6-minute walking distance
BNP: B-type natriuretic peptide
CHF: Chronic heart failure
CI: Confidence intervals
EBCR: Exercise-based cardiac rehabilitation
IQR: Interquartile range
LVEF: Left ventricular ejection fraction
MD: Mean difference
MLHFQ: Minnesota Living with Heart Failure Questionnaire
NYHA: New York Heart Association
RCT: Randomized controlled trial
RM: Routine management
SD: Standard deviation
SMD: Standardized mean difference
TQP: Tai Chi and Qigong Practice
VO_2peak_: Peak oxygen consumption.

## Supplementary Materials

Supplementary Materials eTable 1: sensitivity analysis of the effect size of Peak VO2, 6MWD, MLHFQ, LVEF, and BNP. eFigure 1: flow chart of study identification and selection. eFigure 2: MLHFQ total scores, TQPs plus RMs versus RMs (supplements to Figure 2(a)). eFigure 3: MLHFQ total scores, TQPs plus RMs versus general exercises plus RMs (supplements to Figure 2(b)). eFigure 4: 6MWD (meters), TQPs plus RMs versus RMs (supplements to Figure 3(a)). eFigure 5: 6MWD (meters), TQPs plus RMs versus general exercises plus RMs (supplements to Figure 3(b)). eFigure 6: Peak VO2 (mL/kg/min), TQPs plus RMs versus RMs (supplements to Figure 4(a)). eFigure 7: Peak VO2 (mL/kg/min), TQPs plus RMs versus general exercises plus RMs (supplements to Figure 4(b)). eFigure 8: LVEF (%), TQPs plus RMs versus RMs (supplements to Figure 5(a)). eFigure 9: LVEF (%), TQPs plus RMs versus general exercises plus RMs (supplements to Figure 5(b)). eFigure 10: BNP (pg/mL), TQPs plus RMs versus RMs (supplements to Figure 6(a)). eFigure 11: BNP (pg/mL), TQPs plus RMs versus general exercises plus RMs (supplements to Figure 6(b)). eFigure 12: risk-of-bias summary: eFigure 13: risk-of-bias graph. eFigure 14: publication bias.
