# Supplementary material for: Tai Chi and Qigong Practices for Chronic Heart Failure: A Systematic Review and Meta-Analysis of Randomized Controlled Trials
Source: Evid Based Complement Alternat Med. 2020 Dec 14;2020:2034625. doi: 10.1155/2020/2034625 (PMC7755480; doi:10.1155/2020/2034625)
Supplement: Supplementary Materials — eTable 1: sensitivity analysis of the effect size of Peak VO2, 6MWD, MLHFQ, LVEF, and BNP. eFigure 1: flow chart of study identification and selection. eFigure 2: MLHFQ total scores, TQPs plus RMs versus RMs (supplements to Figure 2(a)). eFigure 3: MLHFQ total scores, TQPs plus RMs versus general exercises plus RMs (supplements to Figure 2(b)). eFigure 4: 6MWD (meters), TQPs plus RMs versus RMs (supplements to Figure 3(a)). eFigure 5: 6MWD (meters), TQPs plus RMs versus general exercises plus RMs (supplements to Figure 3(b)). eFigure 6: Peak VO2 (mL/kg/min), TQPs plus RMs versus RMs (supplements to Figure 4(a)). eFigure 7: Peak VO2 (mL/kg/min), TQPs plus RMs versus general exercises plus RMs (supplements to Figure 4(b)). eFigure 8: LVEF (%), TQPs plus RMs versus RMs (supplements to Figure 5(a)). eFigure 9: LVEF (%), TQPs plus RMs versus general exercises plus RMs (supplements to Figure 5(b)). eFigure 10: BNP (pg/mL), TQPs plus RMs versus RMs (supplements to Figure 6(a)). eFigure 11: BNP (pg/mL), TQPs plus RMs versus general exercises plus RMs (supplements to Figure 6(b)). eFigure 12: risk-of-bias summary: eFigure 13: risk-of-bias graph. eFigure 14: publication bias. [file 2034625.f1.pdf]

Supplementary material

Supplementary to:　Xiankun Chen et al. Taichi and Qigong Practices for Chronic Heart Failure: A Systematic Review and Meta-analysis of Randomized Controlled Trials.

Corresponding authors:

Weihui Lu, Ph.D.

Department of Cardiology

Guangdong Provincial Hospital of Chinese Medicine

111 Dade Road, Guangzhou, Guangdong Province,

510120 Guangzhou, China

Tel:+8620-87351238

E-mail: weihui.lu@163.com

Contents

Search strategy 3

Supplementary Tables 4

eTable 1 Sensitivity analysis of the effect size of Peak VO_2, 6MWD, MLHFQ, LVEF, BNP 4

Supplementary Figures: Flow chart 7

eFig. 1 Flow chart of study identification and selection 7

Supplementary Figures: Meta-analysis results 8

eFig. 2 MLHFQ total scores, TQPs plus RMs versus RMs (supplements to Fig. 2A) **8**

eFig. 3 MLHFQ total scores, TQPs plus RMs versus general exercises plus RMs (supplements to Fig. 2B) **9**

eFig. 4 6MWD (meters), TQPs plus RMs versus RMs (supplements to Fig. 3A) **10**

eFig. 5 6MWD (meters), TQPs plus RMs versus general exercises plus RMs (supplements to Fig. 3B) **11**

eFig. 6 Peak VO2 (mL/kg/min), TQPs plus RMs versus RMs (supplements to Fig. 4A) **12**

eFig. 7 Peak VO2 (mL/kg/min), TQPs plus RMs versus general exercises plus RMs (supplements to Fig. 4B) **12**

eFig. 8 LVEF (%), TQPs plus RMs versus RMs (supplements to Fig. 5A) **13**

eFig. 9 LVEF (%), TQPs plus RMs versus general exercises plus RMs (supplements to Fig. 5B) **14**

eFig. 10 BNP (pg/mL), TQPs plus RMs versus RMs (supplements to Fig. 6A) **15**

eFig. 11 BNP (pg/mL), TQPs plus RMs versus general exercises plus RMs (supplements to Fig. 6B) **16**

Supplementary Figures: Risk of bias and publication bias 17

eFig. 12 Risk of bias summary: 17

eFig. 13 Risk-of-bias graph 17

eFig. 14 Publication bias 18

PRISMA 2009 Checklist 17

Search strategy

PubMed search strategy

1. "Heart Failure"[Mesh] OR
2. “heart failure”[Title/Abstract] OR “cardiac failure”[Title/Abstract] OR “Myocardia failure”[Title/Abstract] OR “Ventricular failure”[Title/Abstract] OR “Heart decompensation”[Title/Abstract]
3. or1/2
4. "traditional exercise*"[Text Word] OR "chinese traditional exercise*" [Text Word] OR "traditional chinese exercise*"[Text Word] OR "chinese exercise*"[Text Word]
5. “taiji”[Title/Abstract] OR “tai ji”[Title/Abstract] OR “tai chi”[Title/Abstract] OR “qigong”[Title/Abstract] OR “qi gong”[Title/Abstract] OR “liuzijue”[Title/Abstract] OR “liu zi jue”[Title/Abstract] OR “wuqinxi”[Title/Abstract] OR “wu qin xi”[Title/Abstract] OR “yijinjing”[Title/Abstract] OR “yi jin jing”[Title/Abstract] OR “baduanjin”[Title/Abstract] OR “ba duan jin”[Title/Abstract] OR “eight section brocades”[Title/Abstract] OR “eight section brocades”[Title/Abstract]
6. or/4-5
7. "Randomized Controlled Trial" [Publication Type]
8. "Controlled Clinical Trial" [Publication Type]
9. randomized[Text Word]
10. placebo[Text Word]
11. randomly[Text Word]
12. trial[Text Word]
13. groups[Text Word]
14. or/7-13
15. ("Animals"[Mesh]) NOT (("Animals"[Mesh]) AND "Humans"[Mesh])
16. 14 not 15
17. 3 AND 6 AND 16

CNKI search strategy

1. FT=随机 OR FT=随机分配 OR FT=随机对照 OR FT=对照 OR FT=盲法 OR FT=单盲 OR FT=双盲 OR FT=随机对照试验 OR FT=随机对 照研究 OR FT=临床试验 OR FT=临床观察 OR FT=临床研究
2. SU=太极拳 OR SU=气功 OR SU=六字诀 OR SU=易筋经 OR SU=五禽戏 OR SU=八段锦 OR SU=传统训练 OR SU=中国传统运动训练 OR SU=中国训练(精确匹配)
3. SU=心血管疾病 OR SU=冠心病 OR SU=高血压 OR SU=高血脂 OR SU=心绞痛 OR SU=心肌梗死 OR SU=心律失常 OR SU=心衰 OR SU=心力衰竭 OR SU=心肌缺血 OR SU=动脉粥样硬化 OR SU=冠状动脉疾病 OR SU=高血压
4. AND/1-

Supplementary Tables

eTable 1 Sensitivity analysis of the effect size of Peak VO_2, 6MWD, MLHFQ, LVEF, BNP

**Outcomes**		**Effect size**		**Heterogeneity**
ID	Study removed	Mean difference [95%CI]	(P-value)	(P-value for I^2); I^2
**MLHFQ, A. TQPs versus no exercise**				
**Overall pooled results**		**-8.63 [-10.60, -6.67]**	**(P < 0.00001)**	**(P < 0.00001); I^2 = 94%**
1	Barrow 2007	-8.47 [-10.46, -6.47]	(P < 0.00001)	(P < 0.00001); I^2 = 94%
3	Chen 2017	-8.88 [-10.91, -6.85]	(P < 0.00001)	(P < 0.00001); I^2 = 94%
4	Feng 2017	-8.48 [-10.52, -6.44]	(P < 0.00001)	(P < 0.00001); I^2 = 94%
5	Huang 2014	-9.17 [-11.19, -7.15]	(P < 0.00001)	(P < 0.00001); I^2 = 94%
10	Sang 2015	-8.28 [-10.24, -6.33]	(P < 0.00001)	(P < 0.00001); I^2 = 90%
12	Xiong 2016	-8.85 [-11.15, -6.55]	(P < 0.00001)	(P < 0.00001); I^2 = 94%
16	Yao 2010	-8.31 [-10.34, -6.28]	(P < 0.00001)	(P < 0.00001); I^2 = 94%
17	Yeh 2004	-8.42 [-10.38, -6.46]	(P < 0.00001)	(P < 0.00001); I^2 = 94%
19	Yeh 2011	-8.49 [-10.49, -6.50]	(P < 0.00001)	(P < 0.00001); I^2 = 94%
22	Yuan 2016	-9.05 [-11.17, -6.94]	(P < 0.00001)	(P < 0.00001); I^2 = 93%
23.1	Zheng 201701 (C1)	-8.18 [-10.15, -6.22]	(P < 0.00001)	(P < 0.00001); I^2 = 94%
24	Shi 2018	-8.29 [-10.33, -6.26]	(P < 0.00001)	(P < 0.00001); I^2 = 94%
26	Liu 2017	-9.01 [-11.34, -6.68]	(P < 0.00001)	(P < 0.00001); I^2 = 94%
32	Lu 2019	-9.06 [-10.79, -7.33]	(P < 0.00001)	(P < 0.00001); I^2 = 89%
**MLHFQ, B. TQPs versus conventional exercises**				
**Overall pooled results**		**-9.18 [-17.95, -0.41]**	**(P = 0.04)**	**(P = 0.0001); I^2 = 86%**
7	Li 2017	-5.63 [-11.76, 0.51]	(P = 0.07)	(P = 0.12); I^2 = 53%
20	Yeh 2013	-10.44 [-20.12, -0.77]	(P = 0.03)	(P < 0.0001); I^2 = 90%
23.2	Zheng 201702 (C2)	-8.29 [-20.69, 4.12]	(P = 0.19)	(P < 0.0001); I^2 = 90%
25	Yu 2018	-13.02 [-20.57, -5.47]	(P = 0.0007)	(P = 0.12); I^2 = 53%
**6MWD, A. TQPs versus no exercise**				
**Overall pooled results**		**59.63 [43.35, 75.90]**	**(P < 0.00001)**	**(P < 0.00001); I^2 = 88%**
5	Huang 2014	65.04 [48.40, 81.68]	(P < 0.00001)	(P < 0.00001); I^2 = 85%
6	Li 2015	58.43 [41.46, 75.41]	(P < 0.00001)	(P < 0.00001); I^2 = 88%
8	Pan 2016	61.22 [43.00, 79.44]	(P < 0.00001)	(P < 0.00001); I^2 = 89%
10	Sang 2015	61.35 [44.32, 78.38]	(P < 0.00001)	(P < 0.00001); I^2 = 89%
12	Xiong 2016	58.29 [41.35, 75.23]	(P < 0.00001)	(P < 0.00001); I^2 = 88%

Outcomes		Effect size		Heterogeneity
ID	Study removed	Mean difference [95%CI]	(P-value)	(P-value for I²); I²
14	Yan 2016	58.41 [41.84, 74.98]	(P < 0.00001)	(P < 0.00001); I² = 89%
15	Yang 2015	58.31 [41.76, 74.86]	(P < 0.00001)	(P < 0.00001); I² = 89%
16	Yao 2010	57.11 [40.55, 73.68]	(P < 0.00001)	(P < 0.00001); I² = 88%
17	Yeh 2004	58.33 [41.94, 74.72]	(P < 0.00001)	(P < 0.00001); I² = 89%
19	Yeh 2011	57.52 [41.87, 73.18]	(P < 0.00001)	(P < 0.00001); I² = 88%
21	Yu 2015	54.84 [39.36, 70.32]	(P < 0.00001)	(P < 0.00001); I² = 88%
23.1	Zheng 201701 (C1)	55.37 [39.89, 70.85]	(P < 0.00001)	(P < 0.00001); I² = 88%
26	Liu 2017	59.54 [39.07, 80.01]	(P < 0.00001)	(P < 0.00001); I² = 88%
30	Yu 2019 (2)	57.48 [40.64, 74.33]	(P < 0.00001)	(P < 0.00001); I² = 88%
31	Deng 2018	49.41 [36.34, 62.48]	(P < 0.00001)	(P < 0.00001); I² = 80%
34.1	Redwine 2019 (C1)	59.03 [43.50, 74.56]	(P < 0.00001)	(P < 0.00001); I² = 88%
35	Zheng 2018	58.11 [42.30, 73.91]	(P < 0.00001)	(P < 0.00001); I² = 88%
**6MWD, B. TQPs versus conventional exercises**				
**Overall pooled results**		**46.66 [-18.17, 111.49]**	(P = 0.16)	(P < 0.00001); I² = 97%
2	Caminiti 2011	51.40 [-30.13, 132.93]	(P = 0.22)	(P < 0.00001); I² = 98%
7	Li 2017	20.86 [7.40, 34.32]	(P = 0.002)	(P = 0.28); I² = 20%
20	Yeh 2013	46.83 [-20.55, 114.22]	(P = 0.17)	(P < 0.00001); I² = 98%
23.2	Zheng 201702 (C2)	42.58 [-27.89, 113.06]	(P = 0.24)	(P < 0.00001); I² = 98%
25	Yu 2018	50.74 [-33.99, 135.48]	(P = 0.24)	(P < 0.00001); I² = 97%
29	Yu 201900	50.29 [-25.56, 126.13]	(P = 0.19)	(P < 0.00001); I² = 98%
34.2	Redwine 2019 (C2)	62.82 [-6.91, 132.55]	(P = 0.08)	(P < 0.00001); I² = 98%
**Peak VO2, A. TQPs versus no exercise**				
**Overall pooled results**		**1.24 [0.91, 1.57]**	**(P < 0.00001)**	**(P = 0.55); I² = 0%**
4	Feng 2017	1.26 [0.80, 1.71]	(P < 0.00001)	(P = 0.35); I² = 5%
17	Yeh 2004	1.25 [0.91, 1.58]	(P < 0.00001)	(P = 0.35); I² = 5%
19	Yeh 2011	1.29 [0.95, 1.63]	(P < 0.00001)	(P = 0.93); I² = 0%
24	Shi 2018	1.14 [0.68, 1.61]	(P < 0.00001)	(P = 0.42); I² = 0%
**Peak VO2, B. TQPs versus conventional exercises**				
**Overall pooled results**		**0.14 [-0.43, 0.70]**	**(P = 0.64)**	**(P = 0.42); I² = 0%**
NA				
**LVEF, A. TQPs versus no exercise**				
**Overall pooled results**		**3.97 [1.22, 6.72]**	**(P = 0.005)**	**(P < 0.00001); I² = 96%**

Outcomes		Effect size		Heterogeneity
ID	Study removed	Mean difference [95%CI]	(P-value)	(P-value for I²); I²
4	Feng 2017	4.58 [1.72, 7.45]	(P = 0.002)	(P < 0.00001) I² = 96%
5	Huang 2014	4.19 [1.21, 7.17]	(P = 0.006)	(P < 0.00001) I² = 96%
8	Pan 2016	4.11 [1.20, 7.02]	(P = 0.006)	(P < 0.00001) I² = 96%
10	Sang 2015	2.92 [1.07, 4.77]	(P = 0.002)	(P < 0.00001) I² = 88%
12	Xiong 2016	3.93 [0.93, 6.92]	(P = 0.01)	(P < 0.00001) I² = 97%
16	Yao 2010	3.43 [0.49, 6.36]	(P = 0.02)	(P < 0.00001) I² = 96%
23.1	Zheng 201701 (C1)	4.20 [1.42, 6.99]	(P = 0.003)	(P < 0.00001) I² = 96%
24	Shi 2018	4.54 [1.64, 7.43]	(P = 0.002)	(P < 0.00001) I² = 96%
26	Liu 2017	3.74 [-0.32, 7.79]	(P = 0.07)	(P < 0.00001) I² = 96%
30	Yu 2019 (2)	3.81 [0.75, 6.87]	(P = 0.01)	(P < 0.00001) I² = 97%
31	Deng 2018	4.05 [0.97, 7.12]	(P = 0.010)	(P < 0.00001) I² = 96%
**LVEF, B. TQPs versus conventional exercises**				
**Overall pooled results**		**3.17 [-1.25, 7.59]**	**(P = 0.14)**	**(P = 0.60); I² = 0%**
20	Yeh 2013	1.84 [-0.33, 4.01]	(P = 0.10)	(P = 0.60); I² = 0%
23.2	Zheng 201702 (C2)	1.74 [-0.39, 3.88]	(P = 0.11)	(P = 0.55); I² = 0%
25	Yu 2018	1.65 [-1.14, 4.45]	(P = 0.25)	(P = 0.40); I² = 0%
29	Yu 201900	0.72 [-2.11, 3.55]	(P = 0.62)	(P = 0.58); I² = 0%
**BNP, A. TQPs versus no exercise**				
**Overall pooled results**		**-76.12 [-134.61, -17.62]**	**(P = 0.01)**	**(P < 0.00001); I² = 95%**
5	Huang 2014	-89.86 [-154.23, -25.48]	(P = 0.006)	(P < 0.00001); I² = 96%
6	Li 2015	-80.84 [-154.47, -7.21]	(P = 0.03)	(P < 0.00001); I² = 96%
8	Pan 2016	-41.57 [-62.44, -20.70]	(P < 0.0001)	(P = 0.13); I² = 39%
14	Yan 2016	-69.96 [-131.96, -7.96]	(P = 0.03)	(P < 0.00001); I² = 96%
15	Yang 2015	-71.19 [-132.84, -9.55]	(P = 0.02)	(P < 0.00001); I² = 96%
17	Yeh 2004	-75.52 [-135.27, -15.78]	(P = 0.01)	(P < 0.00001); I² = 96%
19	Yeh 2011	-84.45 [-148.96, -19.94]	(P = 0.01)	(P < 0.00001); I² = 96%
28	Li 2019	-84.09 [-156.82, -11.37]	(P = 0.02)	(P < 0.00001); I² = 94%
**BNP, B. TQPs versus conventional exercises**				
**Overall pooled results**		**61.29 [-52.22, 174.79]**	**(P = 0.29)**	**(P = 0.04); I² = 77%**
NA				

Supplementary Figures: Flow chart

eFig. 1 Flow chart of study identification and selection

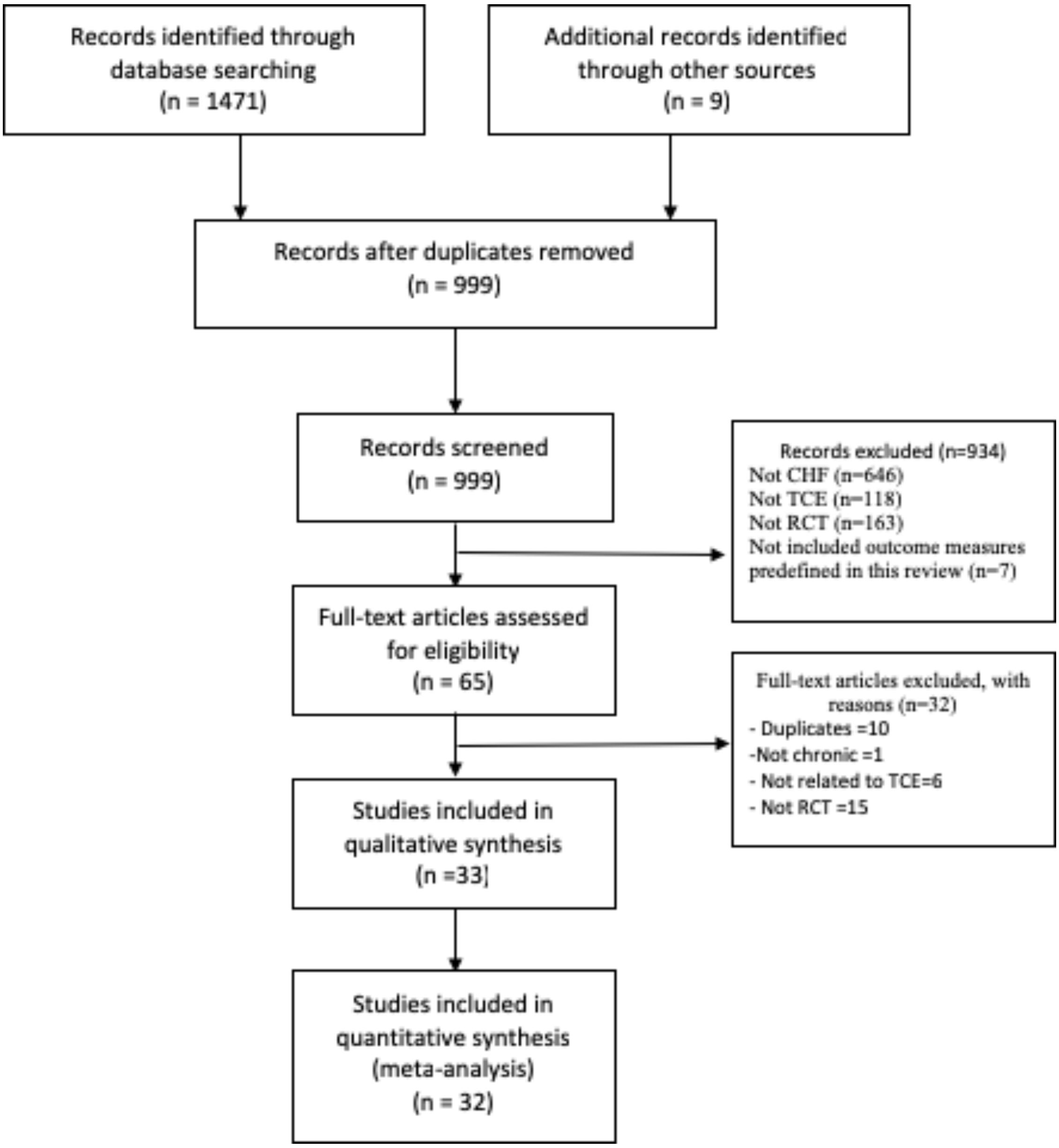

Supplementary Figures: Meta-analysis results

eFig. 2 MLHFQ total scores, TQPs plus RMs versus RMs (supplements to Fig. 2A)

a. The overall pooled results

Study or Subgroup	TQPs Mean	TQPs SD	TQPs Total	Control Mean	Control SD	Control Total	Weight	Mean Difference IV, Random, 95% CI
1. Barrow 2007	18.1	16.6	25	31.6	16.6	27	3.3%	−13.50 [−22.53, −4.47]
3. Chen 2017	15.4	11.74	39	20.33	12.35	41	6.1%	−4.93 [−10.21, 0.35]
4. Feng 2017	25.22	6.97	32	35.96	9.58	28	7.1%	−10.74 [−15.03, −6.45]
5. Huang 2014	38.55	7.41	44	41.02	7.84	44	8.4%	−2.47 [−5.66, 0.72]
10. Sang 2015	32.7	1.9	50	43.2	1.5	50	10.6%	−10.50 [−11.17, −9.83]
12. Xiong 2016	11.2	1.8	33	19.7	1.9	30	10.5%	−8.50 [−9.42, −7.58]
16. Yao 2010	32.6	14.5	80	45.4	12.2	70	7.1%	−12.80 [−17.07, −8.53]
17. Yeh 2004	26	23	15	52	25	15	1.2%	−26.00 [−43.19, −8.81]
19. Yeh 2011	9	17	50	22	29	50	3.1%	−13.00 [−22.32, −3.68]
22. Yuan 2016	37.71	2.37	30	43.04	2.15	30	10.4%	−5.33 [−6.48, −4.18]
23.1 Zheng 2017 (C1)	17.33	1.87	9	38	11.57	7	3.5%	−20.67 [−29.33, −12.01]
24. Shi 2018	56.12	7.63	26	68.72	6.43	29	7.7%	−12.60 [−16.35, −8.85]
26. Liu 2017	35.25	1.75	33	42.52	1.75	33	10.6%	−7.27 [−8.11, −6.43]
32. Lu 2019	9.98	2.56	40	12.58	2.76	40	10.4%	−2.60 [−3.77, −1.43]
**Total (95% CI)**			**506**			**494**	**100.0%**	**−8.63 [−10.60, −6.67]**

Heterogeneity: $Tau^2 = 9.30$; $Chi^2 = 200.44$, df = 13 (P < 0.00001); $I^2 = 94\%$
Test for overall effect: Z = 8.62 (P < 0.00001)

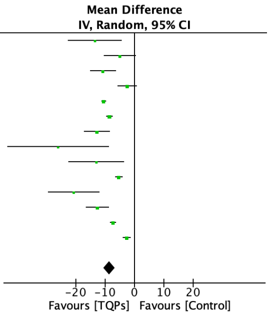

b. Subgroup analysis based on TQPs type

Study or Subgroup	TQPs Mean	TQPs SD	TQPs Total	Control Mean	Control SD	Control Total	Weight	Mean Difference IV, Random, 95% CI
**2.2.1 Tai Chi**								
4. Feng 2017	25.22	6.97	32	35.96	9.58	28	7.1%	−10.74 [−15.03, −6.45]
5. Huang 2014	38.55	7.41	44	41.02	7.84	44	8.4%	−2.47 [−5.66, 0.72]
10. Sang 2015	32.7	1.9	50	43.2	1.5	50	10.6%	−10.50 [−11.17, −9.83]
16. Yao 2010	32.6	14.5	80	45.4	12.2	70	7.1%	−12.80 [−17.07, −8.53]
17. Yeh 2004	26	23	15	52	25	15	1.2%	−26.00 [−43.19, −8.81]
19. Yeh 2011	9	17	50	22	29	50	3.1%	−13.00 [−22.32, −3.68]
22. Yuan 2016	37.71	2.37	30	43.04	2.15	30	10.4%	−5.33 [−6.48, −4.18]
26. Liu 2017	35.25	1.75	33	42.52	1.75	33	10.6%	−7.27 [−8.11, −6.43]
**Subtotal (95% CI)**			**334**			**320**	**58.5%**	**−8.50 [−10.88, −6.13]**
Heterogeneity: $Tau^2 = 7.71$; $Chi^2 = 95.26$, df = 7 (P < 0.00001); $I^2 = 93\%$								
Test for overall effect: Z = 7.02 (P < 0.00001)								
**2.2.2 Qigong**								
3. Chen 2017	15.4	11.74	39	20.33	12.35	41	6.1%	−4.93 [−10.21, 0.35]
12. Xiong 2016	11.2	1.8	33	19.7	1.9	30	10.5%	−8.50 [−9.42, −7.58]
23.1 Zheng 2017 (C1)	17.33	1.87	9	38	11.57	7	3.5%	−20.67 [−29.33, −12.01]
24. Shi 2018	56.12	7.63	26	68.72	6.43	29	7.7%	−12.60 [−16.35, −8.85]
32. Lu 2019	9.98	2.56	40	12.58	2.76	40	10.4%	−2.60 [−3.77, −1.43]
**Subtotal (95% CI)**			**147**			**147**	**38.2%**	**−8.75 [−12.98, −4.52]**
Heterogeneity: $Tau^2 = 18.70$; $Chi^2 = 82.01$, df = 4 (P < 0.00001); $I^2 = 95\%$								
Test for overall effect: Z = 4.06 (P < 0.0001)								
**2.2.3 Tai Chi & Qigong**								
1. Barrow 2007	18.1	16.6	25	31.6	16.6	27	3.3%	−13.50 [−22.53, −4.47]
**Subtotal (95% CI)**			**25**			**27**	**3.3%**	**−13.50 [−22.53, −4.47]**
Heterogeneity: Not applicable								
Test for overall effect: Z = 2.93 (P = 0.003)								
**Total (95% CI)**			**506**			**494**	**100.0%**	**−8.63 [−10.60, −6.67]**

Heterogeneity: $Tau^2 = 9.30$; $Chi^2 = 200.44$, df = 13 (P < 0.00001); $I^2 = 94\%$
Test for overall effect: Z = 8.62 (P < 0.00001)
Test for subgroup differences: $Chi^2 = 1.10$, df = 2 (P = 0.58), $I^2 = 0\%$

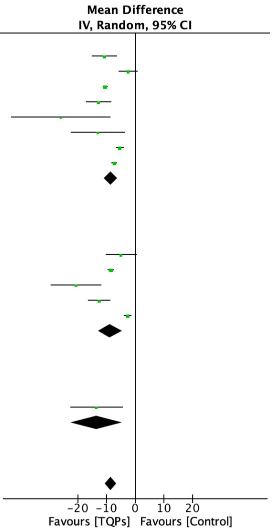

c. Subgroup analysis based on program durations

Study or Subgroup	TQPs Mean	TQPs SD	TQPs Total	Control Mean	Control SD	Control Total	Weight	Mean Difference IV, Random, 95% CI
**2.3.1 4 weeks**								
5. Huang 2014	38.55	7.41	44	41.02	7.84	44	8.4%	−2.47 [−5.66, 0.72]
24. Shi 2018	56.12	7.63	26	68.72	6.43	29	7.7%	−12.60 [−16.35, −8.85]
Subtotal (95% CI)			70			73	16.1%	−7.48 [−17.41, 2.44]
Heterogeneity: $Tau^2 = 48.15$; $Chi^2 = 16.26$, df = 1 (P < 0.0001); $I^2 = 94\%$								
Test for overall effect: Z = 1.48 (P = 0.14)								
**2.3.3 12 weeks**								
3. Chen 2017	15.4	11.74	39	20.33	12.35	41	6.1%	−4.93 [−10.21, 0.35]
10. Sang 2015	32.7	1.9	50	43.2	1.5	50	10.6%	−10.50 [−11.17, −9.83]
12. Xiong 2016	11.2	1.8	33	19.7	1.9	30	10.5%	−8.50 [−9.42, −7.58]
17. Yeh 2004	26	23	15	52	25	15	1.2%	−26.00 [−43.19, −8.81]
19. Yeh 2011	9	17	50	22	29	50	3.1%	−13.00 [−22.32, −3.68]
22. Yuan 2016	37.71	2.37	30	43.04	2.15	30	10.4%	−5.33 [−6.48, −4.18]
23.1 Zheng 2017 (C1)	17.33	1.87	9	38	11.57	7	3.5%	−20.67 [−29.33, −12.01]
26. Liu 2017	35.25	1.75	33	42.52	1.75	33	10.6%	−7.27 [−8.11, −6.43]
Subtotal (95% CI)			259			256	56.0%	−8.66 [−10.69, −6.62]
Heterogeneity: $Tau^2 = 5.05$; $Chi^2 = 85.81$, df = 7 (P < 0.00001); $I^2 = 92\%$								
Test for overall effect: Z = 8.35 (P < 0.00001)								
**2.3.4 16 weeks**								
1. Barrow 2007	18.1	16.6	25	31.6	16.6	27	3.3%	−13.50 [−22.53, −4.47]
Subtotal (95% CI)			25			27	3.3%	−13.50 [−22.53, −4.47]
Heterogeneity: Not applicable								
Test for overall effect: Z = 2.93 (P = 0.003)								
**2.3.5 24 weeks**								
4. Feng 2017	25.22	6.97	32	35.96	9.58	28	7.1%	−10.74 [−15.03, −6.45]
16. Yao 2010	32.6	14.5	80	45.4	12.2	70	7.1%	−12.80 [−17.07, −8.53]
Subtotal (95% CI)			112			98	14.2%	−11.77 [−14.80, −8.75]
Heterogeneity: $Tau^2 = 0.00$; $Chi^2 = 0.44$, df = 1 (P = 0.51); $I^2 = 0\%$								
Test for overall effect: Z = 7.62 (P < 0.00001)								
**2.3.6 52 weeks**								
32. Lu 2019	9.98	2.56	40	12.58	2.76	40	10.4%	−2.60 [−3.77, −1.43]
Subtotal (95% CI)			40			40	10.4%	−2.60 [−3.77, −1.43]
Heterogeneity: Not applicable								
Test for overall effect: Z = 4.37 (P < 0.0001)								
Total (95% CI)			506			494	100.0%	−8.63 [−10.60, −6.67]

Heterogeneity: $Tau^2 = 9.30$; $Chi^2 = 200.44$, df = 13 (P < 0.00001); $I^2 = 94\%$
Test for overall effect: Z = 8.62 (P < 0.00001)
Test for subgroup differences: $Chi^2 = 51.56$, df = 4 (P < 0.00001), $I^2 = 92.2\%$

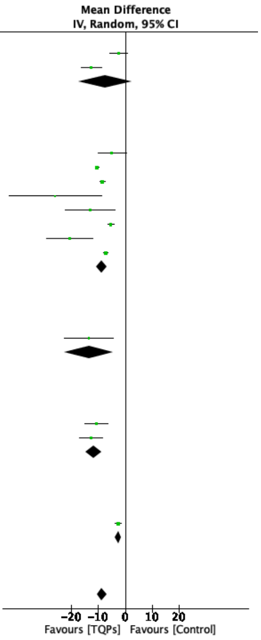

eFig. 3 MLHFQ total scores, TQPs plus RMs versus general exercises plus RMs (supplements to Fig. 2B)

a. The overall pooled results

Study or Subgroup	TQPs Mean	TQPs SD	TQPs Total	Control Mean	Control SD	Control Total	Weight	Mean Difference IV, Random, 95% CI
7. Li 2017	41.27	11.46	30	59.38	12.61	30	28.7%	-18.11 [-24.21, -12.01]
20. Yeh 2013	28.7	16	8	28.6	25	8	11.8%	0.10 [-20.47, 20.67]
23.2 Zheng 2017 (C2)	17.33	1.87	9	28.25	10.55	9	27.5%	-10.92 [-17.92, -3.92]
25. Yu 2018	27.44	7.91	54	30.56	7.94	55	32.1%	-3.12 [-6.10, -0.14]
**Total (95% CI)**			**101**			**102**	**100.0%**	**-9.18 [-17.95, -0.41]**

Heterogeneity: Tau² = 60.12; Chi² = 20.81, df = 3 (P = 0.0001); I² = 86%
Test for overall effect: Z = 2.05 (P = 0.04)

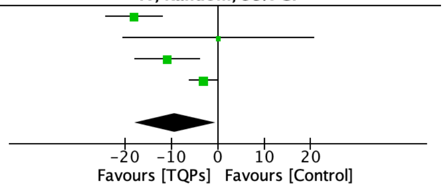

b. Subgroup analysis based on TQPs type

Study or Subgroup	TQPs Mean	TQPs SD	TQPs Total	Control Mean	Control SD	Control Total	Weight	Mean Difference IV, Random, 95% CI
**2.5.1 Tai Chi**								
20. Yeh 2013	28.7	16	8	28.6	25	8	11.8%	0.10 [-20.47, 20.67]
**Subtotal (95% CI)**			**8**			**8**	**11.8%**	**0.10 [-20.47, 20.67]**
Heterogeneity: Not applicable								
Test for overall effect: Z = 0.01 (P = 0.99)								
**2.5.2 Qigong**								
7. Li 2017	41.27	11.46	30	59.38	12.61	30	28.7%	-18.11 [-24.21, -12.01]
23.2 Zheng 2017 (C2)	17.33	1.87	9	28.25	10.55	9	27.5%	-10.92 [-17.92, -3.92]
25. Yu 2018	27.44	7.91	54	30.56	7.94	55	32.1%	-3.12 [-6.10, -0.14]
**Subtotal (95% CI)**			**93**			**94**	**88.2%**	**-10.44 [-20.12, -0.77]**
Heterogeneity: Tau² = 65.16; Chi² = 20.41, df = 2 (P < 0.0001); I² = 90%								
Test for overall effect: Z = 2.12 (P = 0.03)								
**Total (95% CI)**			**101**			**102**	**100.0%**	**-9.18 [-17.95, -0.41]**

Heterogeneity: Tau² = 60.12; Chi² = 20.81, df = 3 (P = 0.0001); I² = 86%
Test for overall effect: Z = 2.05 (P = 0.04)
Test for subgroup differences: Chi² = 0.83, df = 1 (P = 0.36), I² = 0%

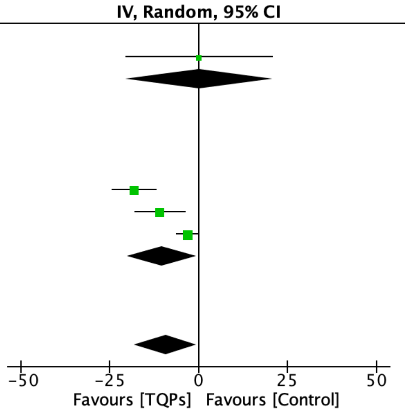

c. Subgroup analysis based on program durations

Study or Subgroup	TQPs Mean	TQPs SD	TQPs Total	Control Mean	Control SD	Control Total	Weight	Mean Difference IV, Random, 95% CI
**2.6.2 8 weeks**								
7. Li 2017	41.27	11.46	30	59.38	12.61	30	28.7%	-18.11 [-24.21, -12.01]
**Subtotal (95% CI)**			**30**			**30**	**28.7%**	**-18.11 [-24.21, -12.01]**
Heterogeneity: Not applicable								
Test for overall effect: Z = 5.82 (P < 0.00001)								
**2.6.3 12 weeks**								
20. Yeh 2013	28.7	16	8	28.6	25	8	11.8%	0.10 [-20.47, 20.67]
23.2 Zheng 2017 (C2)	17.33	1.87	9	28.25	10.55	9	27.5%	-10.92 [-17.92, -3.92]
25. Yu 2018	27.44	7.91	54	30.56	7.94	55	32.1%	-3.12 [-6.10, -0.14]
**Subtotal (95% CI)**			**71**			**72**	**71.3%**	**-5.63 [-11.76, 0.51]**
Heterogeneity: Tau² = 14.92; Chi² = 4.21, df = 2 (P = 0.12); I² = 53%								
Test for overall effect: Z = 1.80 (P = 0.07)								
**Total (95% CI)**			**101**			**102**	**100.0%**	**-9.18 [-17.95, -0.41]**

Heterogeneity: Tau² = 60.12; Chi² = 20.81, df = 3 (P = 0.0001); I² = 86%
Test for overall effect: Z = 2.05 (P = 0.04)
Test for subgroup differences: Chi² = 8.01, df = 1 (P = 0.005), I² = 87.5%

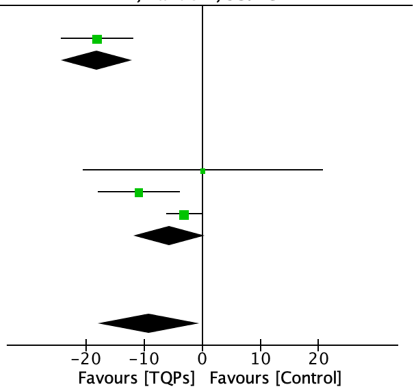

eFig. 4 6MWD (meters), TQPs plus RMs versus RMs (supplements to Fig. 3A)

a. The overall pooled results

Study or Subgroup	TCEs Mean	TCEs SD	TCEs Total	Control Mean	Control SD	Control Total	Weight	Mean Difference IV, Random, 95% CI
5. Huang 2014	368.23	35.7	44	367.7	29.1	44	8.5%	0.53 [−13.08, 14.14]
6. Li 2015	409.6	89.2	90	334.8	84.5	90	7.4%	74.80 [49.42, 100.18]
8. Pan 2016	415	38	31	369	22	30	8.3%	46.00 [30.48, 61.52]
10. Sang 2015	439.78	80.2	50	405.6	110.6	50	6.1%	34.18 [−3.69, 72.05]
12. Xiong 2016	512.5	50.2	33	436.2	49.8	30	7.5%	76.30 [51.58, 101.02]
14. Yan 2016	413.58153	153.54	36	322.1	128.4	36	3.7%	91.48 [26.10, 156.86]
15. Yang 2015	409.67	139.22	30	314.8	124.5	30	3.6%	94.87 [28.04, 161.70]
16. Yao 2010	554	94	80	461	102	70	6.7%	93.00 [61.45, 124.55]
17. Yeh 2004	412	116	15	289	165	15	2.0%	123.00 [20.93, 225.07]
19. Yeh 2011	426	164	50	394	136.3	50	4.1%	32.00 [−27.11, 91.11]
21. Yu 2015	412.56	138.26	41	315.3	123.5	41	4.3%	97.26 [40.51, 154.01]
23.1 Zheng 2017 (C1)	445.77	46.67	9	352.8	85	7	3.4%	92.97 [23.01, 162.93]
26. Liu 2017	314.56	7.82	33	277.4	5	33	9.0%	37.16 [33.99, 40.33]
30. Yu 201902	398.57	41.9	49	349.1	36.3	49	8.3%	49.47 [33.95, 64.99]
31. Deng 2018	558.5	67.1	55	430.8	57.6	56	7.6%	127.70 [104.42, 150.98]
34.1 Redwine 2019 (C1)	237	109	25	246	102	23	4.1%	−9.00 [−68.69, 50.69]
35. Zheng 2018	330.72	105.9	47	302.9	105.9	44	5.5%	27.82 [−15.72, 71.36]
**Total (95% CI)**			**718**			**698**	**100.0%**	**59.63 [43.35, 75.90]**

Heterogeneity: $Tau^2 = 765.09$; $Chi^2 = 134.79$, df = 16 (P < 0.00001); $I^2 = 88\%$
Test for overall effect: Z = 7.18 (P < 0.00001)

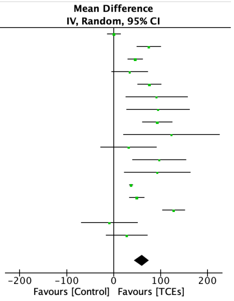

b. Subgroup analysis based on TQPs type

Study or Subgroup	TQPs Mean	TQPs SD	TQPs Total	Control Mean	Control SD	Control Total	Weight	Mean Difference IV, Random, 95% CI
**3.2.1 Tai Chi**								
5. Huang 2014	368.23	35.7	44	367.7	29.1	44	8.5%	0.53 [−13.08, 14.14]
8. Pan 2016	415	38	31	369	22	30	8.3%	46.00 [30.48, 61.52]
10. Sang 2015	439.78	80.2	50	405.6	110.6	50	6.1%	34.18 [−3.69, 72.05]
16. Yao 2010	554	94	80	461	102	70	6.7%	93.00 [61.45, 124.55]
17. Yeh 2004	412	116	15	289	165	15	2.0%	123.00 [20.93, 225.07]
19. Yeh 2011	426	164	50	394	136.3	50	4.1%	32.00 [−27.11, 91.11]
26. Liu 2017	314.56	7.82	33	277.4	5	33	9.0%	37.16 [33.99, 40.33]
31. Deng 2018	558.5	67.1	55	430.8	57.6	56	7.6%	127.70 [104.42, 150.98]
34.1 Redwine 2019 (C1)	237	109	25	246	102	23	4.1%	−9.00 [−68.69, 50.69]
Subtotal (95% CI)			383			371	56.4%	50.99 [27.01, 74.97]
Heterogeneity: $Tau^2 = 985.74$; $Chi^2 = 104.18$, df = 8 (P < 0.00001); $I^2 = 92\%$								
Test for overall effect: Z = 4.17 (P < 0.0001)								
**3.2.2 Qigong**								
6. Li 2015	409.6	89.2	90	334.8	84.5	90	7.4%	74.80 [49.42, 100.18]
12. Xiong 2016	512.5	50.2	33	436.2	49.8	30	7.5%	76.30 [51.58, 101.02]
14. Yan 2016	413.58153	153.54	36	322.1	128.4	36	3.7%	91.48 [26.10, 156.86]
15. Yang 2015	409.67	139.22	30	314.8	124.5	30	3.6%	94.87 [28.04, 161.70]
21. Yu 2015	412.56	138.26	41	315.3	123.5	41	4.3%	97.26 [40.51, 154.01]
23.1 Zheng 2017 (C1)	445.77	46.67	9	352.8	85	7	3.4%	92.97 [23.01, 162.93]
35. Zheng 2018	330.72	105.9	47	302.9	105.9	44	5.5%	27.82 [−15.72, 71.36]
Subtotal (95% CI)			286			278	35.3%	74.11 [59.50, 88.71]
Heterogeneity: $Tau^2 = 0.00$; $Chi^2 = 5.94$, df = 6 (P = 0.43); $I^2 = 0\%$								
Test for overall effect: Z = 9.95 (P < 0.00001)								
**3.2.3 Tai Chi & Qigong**								
30. Yu 201902	398.57	41.9	49	349.1	36.3	49	8.3%	49.47 [33.95, 64.99]
Subtotal (95% CI)			49			49	8.3%	49.47 [33.95, 64.99]
Heterogeneity: Not applicable								
Test for overall effect: Z = 6.25 (P < 0.00001)								
**Total (95% CI)**			718			698	100.0%	59.63 [43.35, 75.90]

Heterogeneity: $Tau^2 = 765.09$; $Chi^2 = 134.79$, df = 16 (P < 0.00001); $I^2 = 88\%$
Test for overall effect: Z = 7.18 (P < 0.00001)
Test for subgroup differences: $Chi^2 = 5.88$, df = 2 (P = 0.05), $I^2 = 66.0\%$

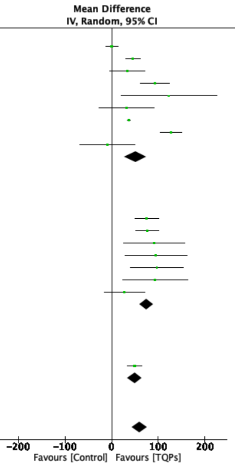

c. Subgroup analysis based on program durations

Study or Subgroup	TQPs Mean	TQPs SD	TQPs Total	Control Mean	Control SD	Control Total	Weight	Mean Difference IV, Random, 95% CI
**3.3.1 4 weeks**								
5. Huang 2014	368.23	35.7	44	367.7	29.1	44	8.5%	0.53 [−13.08, 14.14]
Subtotal (95% CI)			44			44	8.5%	0.53 [−13.08, 14.14]
Heterogeneity: Not applicable								
Test for overall effect: Z = 0.08 (P = 0.94)								
**3.3.3 12 weeks**								
10. Sang 2015	439.78	80.2	50	405.6	110.6	50	6.1%	34.18 [−3.69, 72.05]
12. Xiong 2016	512.5	50.2	33	436.2	49.8	30	7.5%	76.30 [51.58, 101.02]
17. Yeh 2004	412	116	15	289	165	15	2.0%	123.00 [20.93, 225.07]
19. Yeh 2011	426	164	50	394	136.3	50	4.1%	32.00 [−27.11, 91.11]
23.1 Zheng 2017 (C1)	445.77	46.67	9	352.8	85	7	3.4%	92.97 [23.01, 162.93]
26. Liu 2017	314.56	7.82	33	277.4	5	33	9.0%	37.16 [33.99, 40.33]
30. Yu 201902	398.57	41.9	49	349.1	36.3	49	8.3%	49.47 [33.95, 64.99]
35. Zheng 2018	330.72	105.9	47	302.9	105.9	44	5.5%	27.82 [−15.72, 71.36]
Subtotal (95% CI)			286			278	45.8%	48.95 [34.32, 63.59]
Heterogeneity: $Tau^2 = 177.60$; $Chi^2 = 16.87$, df = 7 (P = 0.02); $I^2 = 59\%$								
Test for overall effect: Z = 6.56 (P < 0.00001)								
**3.3.4 16 weeks**								
34.1 Redwine 2019 (C1)	237	109	25	246	102	23	4.1%	−9.00 [−68.69, 50.69]
Subtotal (95% CI)			25			23	4.1%	−9.00 [−68.69, 50.69]
Heterogeneity: Not applicable								
Test for overall effect: Z = 0.30 (P = 0.77)								
**3.3.5 24 weeks**								
8. Pan 2016	415	38	31	369	22	30	8.3%	46.00 [30.48, 61.52]
16. Yao 2010	554	94	80	461	102	70	6.7%	93.00 [61.45, 124.55]
31. Deng 2018	558.5	67.1	55	430.8	57.6	56	7.6%	127.70 [104.42, 150.98]
Subtotal (95% CI)			166			156	22.7%	88.32 [34.00, 142.65]
Heterogeneity: $Tau^2 = 2152.98$; $Chi^2 = 34.33$, df = 2 (P < 0.00001); $I^2 = 94\%$								
Test for overall effect: Z = 3.19 (P = 0.001)								
**3.3.6 52 weeks**								
6. Li 2015	409.6	89.2	90	334.8	84.5	90	7.4%	74.80 [49.42, 100.18]
14. Yan 2016	413.58153	153.54	36	322.1	128.4	36	3.7%	91.48 [26.10, 156.86]
15. Yang 2015	409.67	139.22	30	314.8	124.5	30	3.6%	94.87 [28.04, 161.70]
21. Yu 2015	412.56	138.26	41	315.3	123.5	41	4.3%	97.26 [40.51, 154.01]
Subtotal (95% CI)			197			197	18.9%	81.42 [60.66, 102.18]
Heterogeneity: $Tau^2 = 0.00$; $Chi^2 = 0.81$, df = 3 (P = 0.85); $I^2 = 0\%$								
Test for overall effect: Z = 7.69 (P < 0.00001)								
**Total (95% CI)**			718			698	100.0%	59.63 [43.35, 75.90]

Heterogeneity: $Tau^2 = 765.09$; $Chi^2 = 134.79$, df = 16 (P < 0.00001); $I^2 = 88\%$
Test for overall effect: Z = 7.18 (P < 0.00001)
Test for subgroup differences: $Chi^2 = 53.12$, df = 4 (P < 0.00001), $I^2 = 92.5\%$

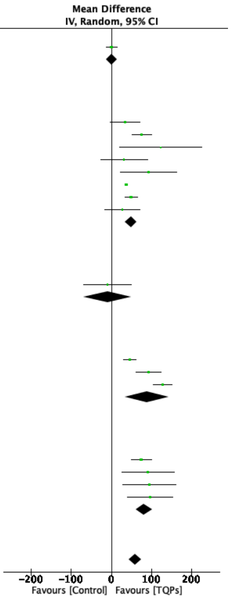

eFig. 5 6MWD (meters), TQPs plus RMs versus general exercises plus RMs (supplements to Fig. 3B)

a. The overall pooled results

Study or Subgroup	TQPs Mean	TQPs SD	TQPs Total	Control Mean	Control SD	Control Total	Weight	Mean Difference IV, Random, 95% CI
2. Caminiti 2011	291.5	46	30	272	33	30	16.5%	19.50 [−0.76, 39.76]
7. Li 2017	550.6	40.51	30	368.71	32.24	30	16.5%	181.89 [163.36, 200.42]
20. Yeh 2013	404.2	190	8	360.1	205	8	6.7%	44.10 [−149.59, 237.79]
23.2 Zheng 2017 (C2)	445.77	46.67	9	372.87	107.85	8	13.3%	72.90 [−7.82, 153.62]
25. Yu 2018	419.61	26.33	54	397.2	39.1	54	16.6%	22.41 [9.84, 34.98]
29. Yu 201900	391.65	86	80	365.3	75.4	40	16.2%	26.35 [−3.67, 56.37]
34.2 Redwine 2019 (C2)	237	109	25	288	124	22	14.2%	−51.00 [−118.16, 16.16]
**Total (95% CI)**			**236**			**192**	**100.0%**	**46.66 [−18.17, 111.49]**

Heterogeneity: Tau^2 = 6534.07; Chi^2 = 230.91, df = 6 (P < 0.00001); I^2 = 97%
Test for overall effect: Z = 1.41 (P = 0.16)

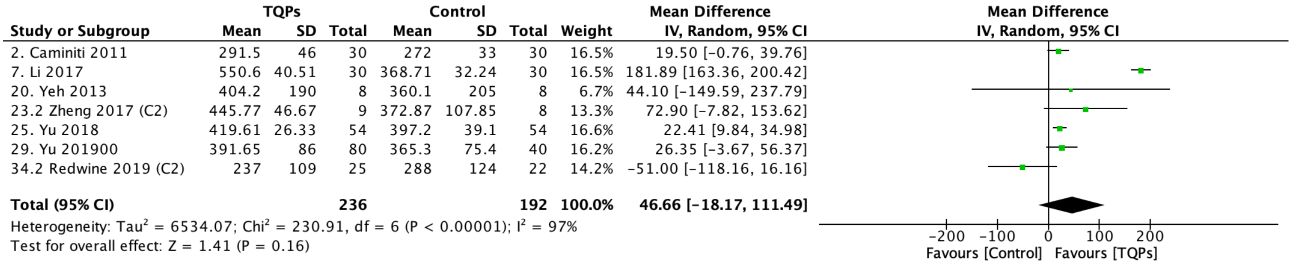

b. Subgroup analysis based on TQPs type

Study or Subgroup	TQPs Mean	TQPs SD	TQPs Total	Control Mean	Control SD	Control Total	Weight	Mean Difference IV, Random, 95% CI
**3.8.1 Tai Chi**								
2. Caminiti 2011	291.5	46	30	272	33	30	16.5%	19.50 [−0.76, 39.76]
20. Yeh 2013	404.2	190	8	360.1	205	8	6.7%	44.10 [−149.59, 237.79]
29. Yu 201900	391.65	86	80	365.3	75.4	40	16.2%	26.35 [−3.67, 56.37]
34.2 Redwine 2019 (C2)	237	109	25	288	124	22	14.2%	−51.00 [−118.16, 16.16]
**Subtotal (95% CI)**			**143**			**100**	**53.5%**	**14.35 [−10.11, 38.81]**
Heterogeneity: Tau^2 = 198.08; Chi^2 = 4.44, df = 3 (P = 0.22); I^2 = 32%								
Test for overall effect: Z = 1.15 (P = 0.25)								
**3.8.2 Qigong**								
7. Li 2017	550.6	40.51	30	368.71	32.24	30	16.5%	181.89 [163.36, 200.42]
25. Yu 2018	419.61	26.33	54	397.2	39.1	54	16.6%	22.41 [9.84, 34.98]
23.2 Zheng 2017 (C2)	445.77	46.67	9	372.87	107.85	8	13.3%	72.90 [−7.82, 153.62]
**Subtotal (95% CI)**			**93**			**92**	**46.5%**	**93.10 [−33.13, 219.34]**
Heterogeneity: Tau^2 = 11881.30; Chi^2 = 194.90, df = 2 (P < 0.00001); I^2 = 99%								
Test for overall effect: Z = 1.45 (P = 0.15)								
**Total (95% CI)**			**236**			**192**	**100.0%**	**46.66 [−18.17, 111.49]**

Heterogeneity: Tau^2 = 6534.07; Chi^2 = 230.91, df = 6 (P < 0.00001); I^2 = 97%
Test for overall effect: Z = 1.41 (P = 0.16)
Test for subgroup differences: Chi^2 = 1.44, df = 1 (P = 0.23), I^2 = 30.6%

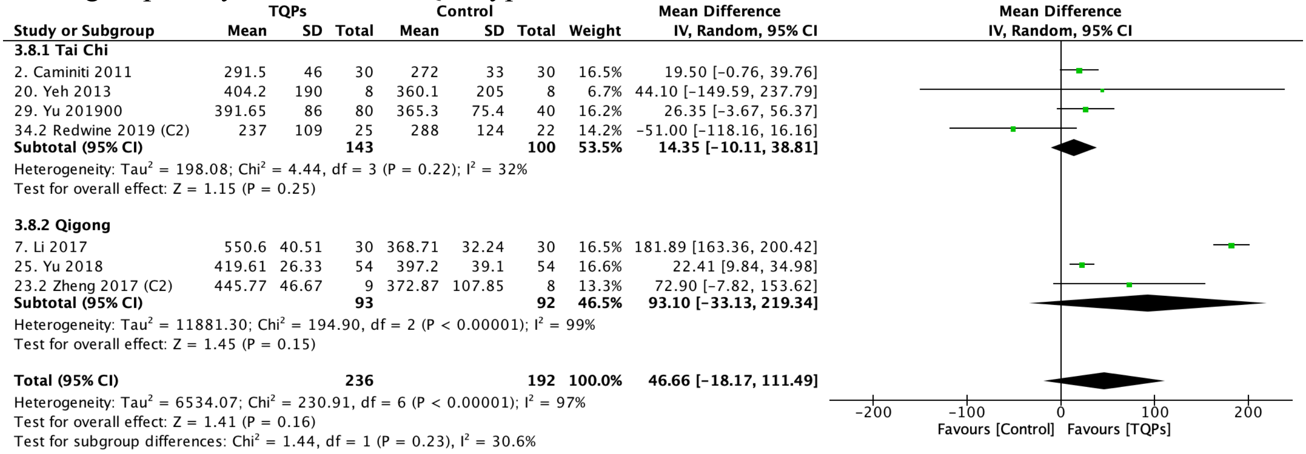

c. Subgroup analysis based on program durations

Study or Subgroup	TQPs Mean	TQPs SD	TQPs Total	Control Mean	Control SD	Control Total	Weight	Mean Difference IV, Random, 95% CI
**3.9.2 8 weeks**								
7. Li 2017	550.6	40.51	25	368.71	32.24	30	16.2%	181.89 [162.26, 201.52]
**Subtotal (95% CI)**			**25**			**30**	**16.2%**	**181.89 [162.26, 201.52]**
Heterogeneity: Not applicable								
Test for overall effect: Z = 18.16 (P < 0.00001)								
**3.9.3 12 weeks**								
2. Caminiti 2011	291.5	46	30	272	33	30	16.2%	19.50 [−0.76, 39.76]
20. Yeh 2013	404.2	190	30	360.1	205	8	8.0%	44.10 [−113.39, 201.59]
23.2 Zheng 2017 (C2)	445.77	46.67	8	372.87	107.85	9	13.1%	72.90 [−4.63, 150.43]
25. Yu 2018	419.61	26.33	54	397.2	39.1	54	16.4%	22.41 [9.84, 34.98]
**Subtotal (95% CI)**			**122**			**101**	**53.7%**	**22.65 [12.09, 33.21]**
Heterogeneity: Tau^2 = 0.00; Chi^2 = 1.78, df = 3 (P = 0.62); I^2 = 0%								
Test for overall effect: Z = 4.21 (P < 0.0001)								
**3.9.4 16 weeks**								
34.2 Redwine 2019 (C2)	237	109	40	288	124	22	14.2%	−51.00 [−112.85, 10.85]
**Subtotal (95% CI)**			**40**			**22**	**14.2%**	**−51.00 [−112.85, 10.85]**
Heterogeneity: Not applicable								
Test for overall effect: Z = 1.62 (P = 0.11)								
**3.9.5 24 weeks**								
29. Yu 201900	391.65	86	80	365.3	75.4	40	15.9%	26.35 [−3.67, 56.37]
**Subtotal (95% CI)**			**80**			**40**	**15.9%**	**26.35 [−3.67, 56.37]**
Heterogeneity: Not applicable								
Test for overall effect: Z = 1.72 (P = 0.09)								
**Total (95% CI)**			**267**			**193**	**100.0%**	**46.38 [−16.02, 108.79]**

Heterogeneity: Tau^2 = 6152.32; Chi^2 = 213.04, df = 6 (P < 0.00001); I^2 = 97%
Test for overall effect: Z = 1.46 (P = 0.15)
Test for subgroup differences: Chi^2 = 211.26, df = 3 (P < 0.00001), I^2 = 98.6%

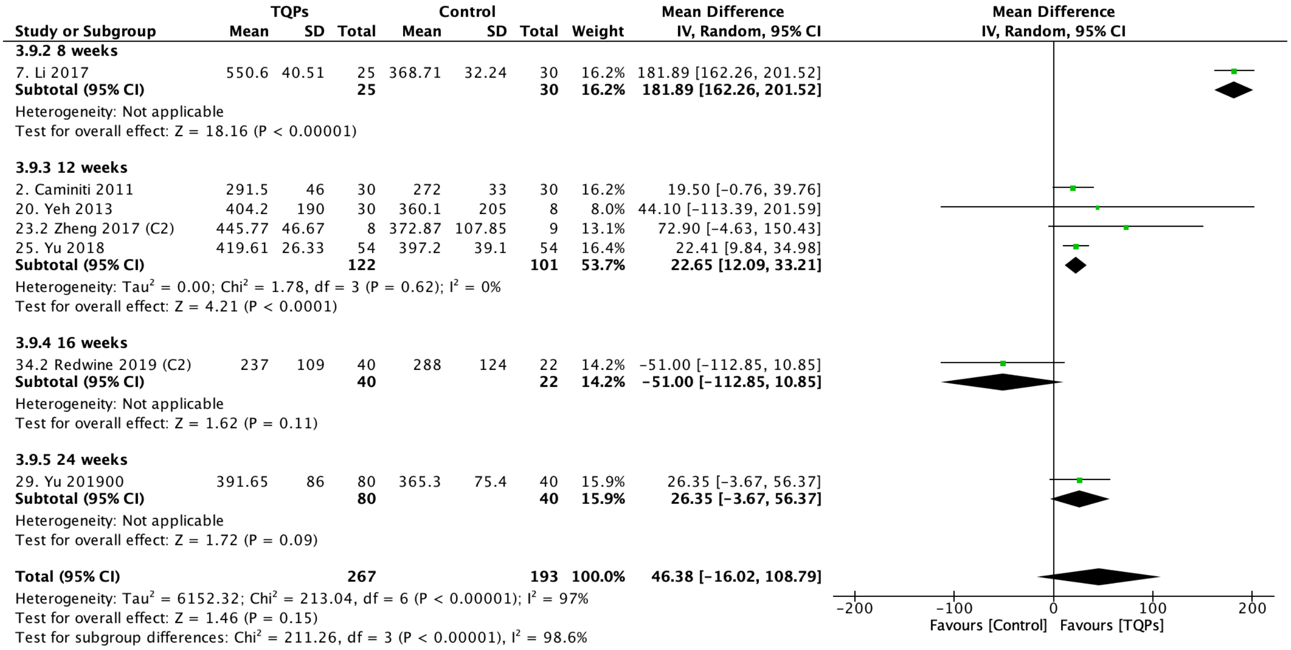

eFig. 6 Peak VO2 (mL/kg/min), TQPs plus RMs versus RMs (supplements to Fig. 4A)

a. The overall pooled results

Study or Subgroup	TQPs Mean	TQPs SD	TQPs Total	Control Mean	Control SD	Control Total	Weight	Mean Difference IV, Fixed, 95% CI
4. Feng 2017	16.72	1.05	32	15.49	0.87	28	46.5%	1.23 [0.74, 1.72]
17. Yeh 2004	11.4	3	15	10.4	6	15	1.0%	1.00 [−2.39, 4.39]
19. Yeh 2011	13	3.7	50	13	5.2	50	3.5%	0.00 [−1.77, 1.77]
24. Shi 2018	15.71	0.86	26	14.36	0.93	29	49.1%	1.35 [0.88, 1.82]
**Total (95% CI)**			**123**			**122**	**100.0%**	**1.24 [0.91, 1.57]**

Heterogeneity: Chi^2 = 2.12, df = 3 (P = 0.55); I^2 = 0%
Test for overall effect: Z = 7.36 (P < 0.00001)

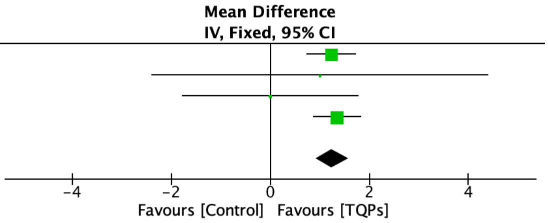

b. Subgroup analysis based on TQPs type

Study or Subgroup	TQPs Mean	TQPs SD	TQPs Total	Control Mean	Control SD	Control Total	Weight	Mean Difference IV, Fixed, 95% CI
**4.2.1 Tai Chi**								
4. Feng 2017	16.72	1.05	32	15.49	0.87	28	46.5%	1.23 [0.74, 1.72]
17. Yeh 2004	11.4	3	15	10.4	6	15	1.0%	1.00 [−2.39, 4.39]
19. Yeh 2011	13	3.7	50	13	5.2	50	3.5%	0.00 [−1.77, 1.77]
**Subtotal (95% CI)**			**97**			**93**	**50.9%**	**1.14 [0.68, 1.61]**
Heterogeneity: Chi^2 = 1.73, df = 2 (P = 0.42); I^2 = 0%								
Test for overall effect: Z = 4.82 (P < 0.00001)								
**4.2.2 Qigong**								
24. Shi 2018	15.71	0.86	26	14.36	0.93	29	49.1%	1.35 [0.88, 1.82]
**Subtotal (95% CI)**			**26**			**29**	**49.1%**	**1.35 [0.88, 1.82]**
Heterogeneity: Not applicable								
Test for overall effect: Z = 5.59 (P < 0.00001)								
**Total (95% CI)**			**123**			**122**	**100.0%**	**1.24 [0.91, 1.57]**

Heterogeneity: Chi^2 = 2.12, df = 3 (P = 0.55); I^2 = 0%
Test for overall effect: Z = 7.36 (P < 0.00001)
Test for subgroup differences: Chi^2 = 0.38, df = 1 (P = 0.54), I^2 = 0%

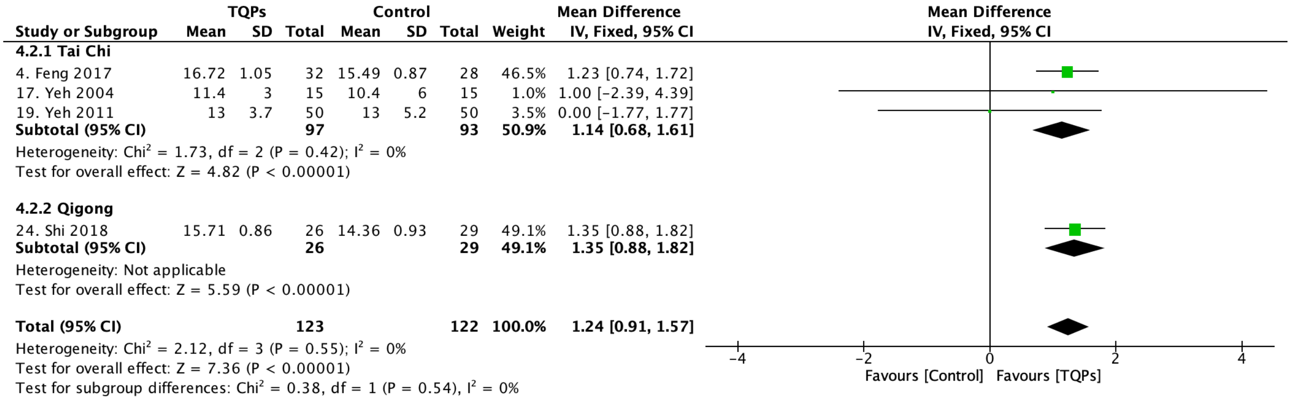

c. Subgroup analysis based on program durations

Study or Subgroup	TQPs Mean	TQPs SD	TQPs Total	Control Mean	Control SD	Control Total	Weight	Mean Difference IV, Fixed, 95% CI
**4.3.1 4 weeks**								
24. Shi 2018	15.71	0.86	26	14.36	0.93	29	49.1%	1.35 [0.88, 1.82]
**Subtotal (95% CI)**			**26**			**29**	**49.1%**	**1.35 [0.88, 1.82]**
Heterogeneity: Not applicable								
Test for overall effect: Z = 5.59 (P < 0.00001)								
**4.3.3 12 weeks**								
17. Yeh 2004	11.4	3	15	10.4	6	15	1.0%	1.00 [−2.39, 4.39]
19. Yeh 2011	13	3.7	50	13	5.2	50	3.5%	0.00 [−1.77, 1.77]
**Subtotal (95% CI)**			**65**			**65**	**4.5%**	**0.21 [−1.36, 1.78]**
Heterogeneity: Chi^2 = 0.26, df = 1 (P = 0.61); I^2 = 0%								
Test for overall effect: Z = 0.27 (P = 0.79)								
**4.3.5 24 weeks**								
4. Feng 2017	16.72	1.05	32	15.49	0.87	28	46.5%	1.23 [0.74, 1.72]
**Subtotal (95% CI)**			**32**			**28**	**46.5%**	**1.23 [0.74, 1.72]**
Heterogeneity: Not applicable								
Test for overall effect: Z = 4.96 (P < 0.00001)								
**Total (95% CI)**			**123**			**122**	**100.0%**	**1.24 [0.91, 1.57]**

Heterogeneity: Chi^2 = 2.12, df = 3 (P = 0.55); I^2 = 0%
Test for overall effect: Z = 7.36 (P < 0.00001)
Test for subgroup differences: Chi^2 = 1.85, df = 2 (P = 0.40), I^2 = 0%

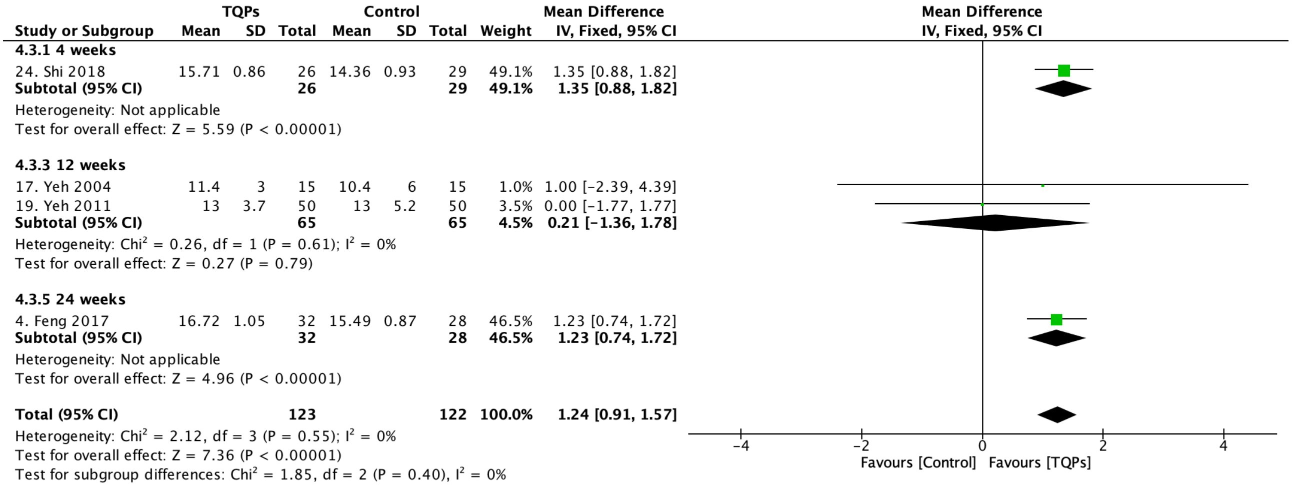

eFig. 7 Peak VO2 (mL/kg/min), TQPs plus RMs versus general exercises plus RMs (supplements to Fig. 4B)

a. The overall pooled results

Study or Subgroup	TQPs Mean	TQPs SD	TQPs Total	Control Mean	Control SD	Control Total	Weight	Mean Difference IV, Fixed, 95% CI
20. Yeh 2013	15.2	6	8	13	4	8	1.3%	2.20 [−2.80, 7.20]
25. Yu 2018	15.54	0.9	54	15.43	1.96	55	98.7%	0.11 [−0.46, 0.68]
**Total (95% CI)**			**62**			**63**	**100.0%**	**0.14 [−0.43, 0.70]**

Heterogeneity: Chi^2 = 0.66, df = 1 (P = 0.42); I^2 = 0%
Test for overall effect: Z = 0.47 (P = 0.64)

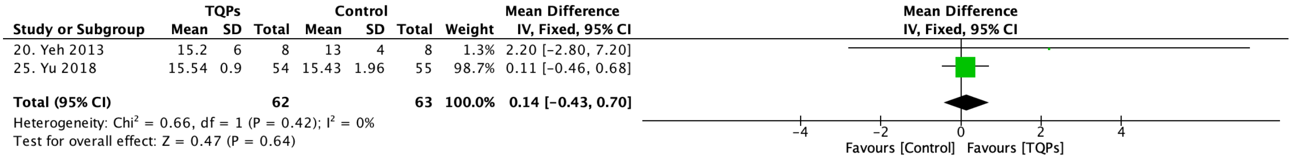

eFig. 8 LVEF (%), TQPs plus RMs versus RMs (supplements to Fig. 5A)

a. The overall pooled results

Study or Subgroup	TQPs Mean	TQPs SD	TQPs Total	Control Mean	Control SD	Control Total	Weight	Mean Difference IV, Random, 95% CI
4. Feng 2017	42	3	32	44	8	28	9.3%	−2.00 [−5.14, 1.14]
5. Huang 2014	49.89	6.26	44	48.14	5.78	44	9.7%	1.75 [−0.77, 4.27]
8. Pan 2016	36.3	9.3	31	33.9	7.9	30	8.4%	2.40 [−1.93, 6.73]
10. Sang 2015	56.2	3.8	50	41.8	3.7	50	10.3%	14.40 [12.93, 15.87]
12. Xiong 2016	48.9	5.8	33	44.7	4.9	30	9.6%	4.20 [1.56, 6.84]
16. Yao 2010	48.63	9.37	80	39.62	7.28	70	9.6%	9.01 [6.34, 11.68]
23.1 Zheng 2017 (C1)	58.78	12.81	9	64.24	17.64	7	2.4%	−5.46 [−20.98, 10.06]
24. Shi 2018	43	3	26	44	3	29	10.2%	−1.00 [−2.59, 0.59]
26. Liu 2017	53.77	0.78	33	49.39	0.69	33	10.5%	4.38 [4.02, 4.74]
30. Yu 201902	51.9	5.52	49	46.77	5.03	49	10.0%	5.13 [3.04, 7.22]
31. Deng 2018	49	5.4	55	46	5.2	56	10.0%	3.00 [1.03, 4.97]
**Total (95% CI)**			**442**			**426**	**100.0%**	**3.97 [1.22, 6.72]**

Heterogeneity: $Tau^2 = 18.70$; $Chi^2 = 257.72$, df = 10 (P < 0.00001); $I^2 = 96\%$
Test for overall effect: Z = 2.83 (P = 0.005)

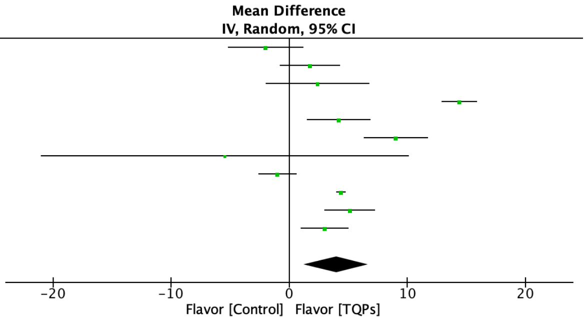

b. Subgroup analysis based on TQPs type

Study or Subgroup	TQPs Mean	TQPs SD	TQPs Total	Control Mean	Control SD	Control Total	Weight	Mean Difference IV, Random, 95% CI
**5.2.1 Tai Chi**								
4. Feng 2017	42	3	32	44	8	28	9.3%	−2.00 [−5.14, 1.14]
5. Huang 2014	49.89	6.26	44	48.14	5.78	44	9.7%	1.75 [−0.77, 4.27]
8. Pan 2016	36.3	9.3	31	33.9	7.9	30	8.4%	2.40 [−1.93, 6.73]
10. Sang 2015	56.2	3.8	50	41.8	3.7	50	10.3%	14.40 [12.93, 15.87]
16. Yao 2010	48.63	9.37	80	39.62	7.28	70	9.6%	9.01 [6.34, 11.68]
26. Liu 2017	53.77	0.78	33	49.39	0.69	33	10.5%	4.38 [4.02, 4.74]
31. Deng 2018	49	5.4	55	46	5.2	56	10.0%	3.00 [1.03, 4.97]
**Subtotal (95% CI)**			**325**			**311**	**67.8%**	**4.82 [1.05, 8.60]**
Heterogeneity: $Tau^2 = 24.23$; $Chi^2 = 206.77$, df = 6 (P < 0.00001); $I^2 = 97\%$								
Test for overall effect: Z = 2.50 (P = 0.01)								
**5.2.2 Qigong**								
12. Xiong 2016	48.9	5.8	33	44.7	4.9	30	9.6%	4.20 [1.56, 6.84]
23.1 Zheng 2017 (C1)	58.78	12.81	9	64.24	17.64	7	2.4%	−5.46 [−20.98, 10.06]
24. Shi 2018	43	3	26	44	3	29	10.2%	−1.00 [−2.59, 0.59]
**Subtotal (95% CI)**			**68**			**66**	**22.3%**	**0.94 [−3.78, 5.65]**
Heterogeneity: $Tau^2 = 11.35$; $Chi^2 = 11.46$, df = 2 (P = 0.003); $I^2 = 83\%$								
Test for overall effect: Z = 0.39 (P = 0.70)								
**5.2.3 Tai Chi & Qigong**								
30. Yu 201902	51.9	5.52	49	46.77	5.03	49	10.0%	5.13 [3.04, 7.22]
**Subtotal (95% CI)**			**49**			**49**	**10.0%**	**5.13 [3.04, 7.22]**
Heterogeneity: Not applicable								
Test for overall effect: Z = 4.81 (P < 0.00001)								
**Total (95% CI)**			**442**			**426**	**100.0%**	**3.97 [1.22, 6.72]**

Heterogeneity: $Tau^2 = 18.70$; $Chi^2 = 257.72$, df = 10 (P < 0.00001); $I^2 = 96\%$
Test for overall effect: Z = 2.83 (P = 0.005)
Test for subgroup differences: $Chi^2 = 2.57$, df = 2 (P = 0.28), $I^2 = 22.1\%$

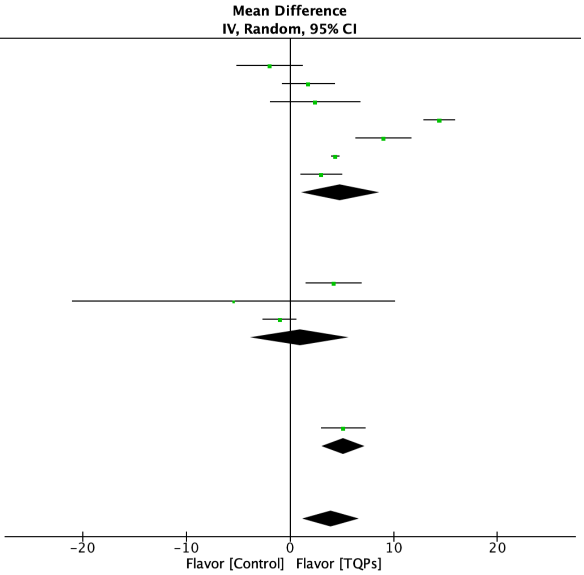

c. Subgroup analysis based on program durations

Study or Subgroup	TQPs Mean	TQPs SD	TQPs Total	Control Mean	Control SD	Control Total	Weight	Mean Difference IV, Random, 95% CI
**5.3.1 4 weeks**								
5. Huang 2014	49.89	6.26	44	48.14	5.78	44	9.7%	1.75 [−0.77, 4.27]
24. Shi 2018	43	3	26	44	3	29	10.2%	−1.00 [−2.59, 0.59]
**Subtotal (95% CI)**			**70**			**73**	**19.9%**	**0.19 [−2.48, 2.87]**
Heterogeneity: $Tau^2 = 2.63$; $Chi^2 = 3.28$, df = 1 (P = 0.07); $I^2 = 70\%$								
Test for overall effect: Z = 0.14 (P = 0.89)								
**5.3.3 12 weeks**								
10. Sang 2015	56.2	3.8	50	41.8	3.7	50	10.3%	14.40 [12.93, 15.87]
12. Xiong 2016	48.9	5.8	33	44.7	4.9	30	9.6%	4.20 [1.56, 6.84]
23.1 Zheng 2017 (C1)	58.78	12.81	9	64.24	17.64	7	2.4%	−5.46 [−20.98, 10.06]
26. Liu 2017	53.77	0.78	33	49.39	0.69	33	10.5%	4.38 [4.02, 4.74]
30. Yu 201902	51.9	5.52	49	46.77	5.03	49	10.0%	5.13 [3.04, 7.22]
**Subtotal (95% CI)**			**174**			**169**	**42.8%**	**6.14 [1.09, 11.18]**
Heterogeneity: $Tau^2 = 27.74$; $Chi^2 = 170.66$, df = 4 (P < 0.00001); $I^2 = 98\%$								
Test for overall effect: Z = 2.38 (P = 0.02)								
**5.3.6 52 weeks**								
4. Feng 2017	42	3	32	44	8	28	9.3%	−2.00 [−5.14, 1.14]
8. Pan 2016	36.3	9.3	31	33.9	7.9	30	8.4%	2.40 [−1.93, 6.73]
16. Yao 2010	48.63	9.37	80	39.62	7.28	70	9.6%	9.01 [6.34, 11.68]
31. Deng 2018	49	5.4	55	46	5.2	56	10.0%	3.00 [1.03, 4.97]
**Subtotal (95% CI)**			**198**			**184**	**37.3%**	**3.17 [−1.25, 7.59]**
Heterogeneity: $Tau^2 = 17.89$; $Chi^2 = 28.64$, df = 3 (P < 0.00001); $I^2 = 90\%$								
Test for overall effect: Z = 1.41 (P = 0.16)								
**Total (95% CI)**			**442**			**426**	**100.0%**	**3.97 [1.22, 6.72]**

Heterogeneity: $Tau^2 = 18.70$; $Chi^2 = 257.72$, df = 10 (P < 0.00001); $I^2 = 96\%$
Test for overall effect: Z = 2.83 (P = 0.005)
Test for subgroup differences: $Chi^2 = 4.59$, df = 2 (P = 0.10), $I^2 = 56.4\%$

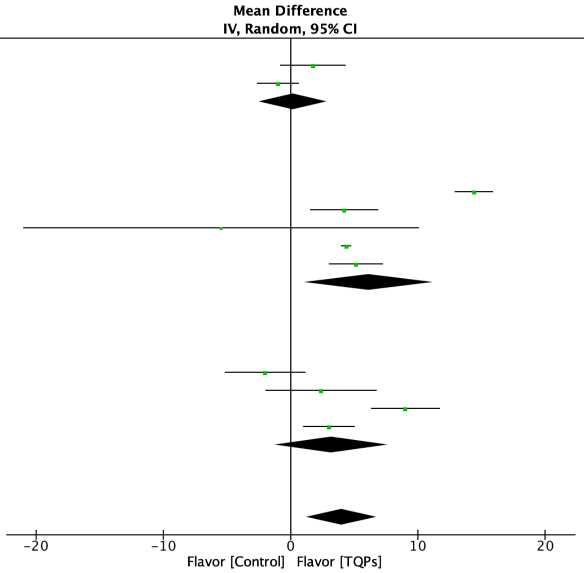

eFig. 9 LVEF (%), TQPs plus RMs versus general exercises plus RMs (supplements to Fig. 5B)

a. The overall pooled results

Study or Subgroup	TQPs Mean	TQPs SD	TQPs Total	Control Mean	Control SD	Control Total	Weight	Mean Difference IV, Fixed, 95% CI
20. Yeh 2013	62	9	8	64	7	8	7.0%	−2.00 [−9.90, 5.90]
23.2 Zheng 2017 (C2)	58.78	12.81	9	61.62	9.62	8	3.8%	−2.84 [−13.54, 7.86]
25. Yu 2018	54.3	8.99	54	52.84	7.79	55	43.9%	1.46 [−1.70, 4.62]
29. Yu 201900	41.4	8.2	80	38.8	8.2	40	45.3%	2.60 [−0.51, 5.71]
**Total (95% CI)**			**151**			**111**	**100.0%**	**1.57 [−0.53, 3.66]**

Heterogeneity: $Chi^2 = 1.86$, df = 3 (P = 0.60); $I^2 = 0\%$
Test for overall effect: Z = 1.47 (P = 0.14)

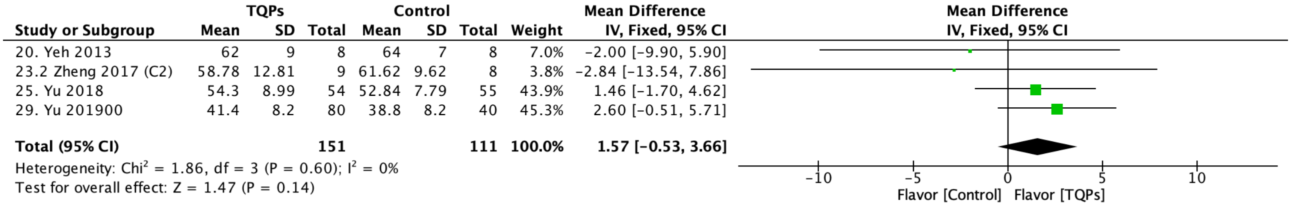

b. Subgroup analysis based on TQPs type

Study or Subgroup	TQPs Mean	TQPs SD	TQPs Total	Control Mean	Control SD	Control Total	Weight	Mean Difference IV, Fixed, 95% CI
**5.5.1 Tai Chi**								
20. Yeh 2013	62	9	8	64	7	8	7.0%	−2.00 [−9.90, 5.90]
29. Yu 201900	41.4	8.2	80	38.8	8.2	40	45.3%	2.60 [−0.51, 5.71]
**Subtotal (95% CI)**			**88**			**48**	**52.3%**	**1.98 [−0.91, 4.88]**
Heterogeneity: $Chi^2 = 1.13$, df = 1 (P = 0.29); $I^2 = 11\%$								
Test for overall effect: Z = 1.34 (P = 0.18)								
**5.5.2 Qigong**								
23.2 Zheng 2017 (C2)	58.78	12.81	9	61.62	9.62	8	3.8%	−2.84 [−13.54, 7.86]
25. Yu 2018	54.3	8.99	54	52.84	7.79	55	43.9%	1.46 [−1.70, 4.62]
**Subtotal (95% CI)**			**63**			**63**	**47.7%**	**1.11 [−1.92, 4.15]**
Heterogeneity: $Chi^2 = 0.57$, df = 1 (P = 0.45); $I^2 = 0\%$								
Test for overall effect: Z = 0.72 (P = 0.47)								
**Total (95% CI)**			**151**			**111**	**100.0%**	**1.57 [−0.53, 3.66]**

Heterogeneity: $Chi^2 = 1.86$, df = 3 (P = 0.60); $I^2 = 0\%$
Test for overall effect: Z = 1.47 (P = 0.14)
Test for subgroup differences: $Chi^2 = 0.16$, df = 1 (P = 0.69), $I^2 = 0\%$

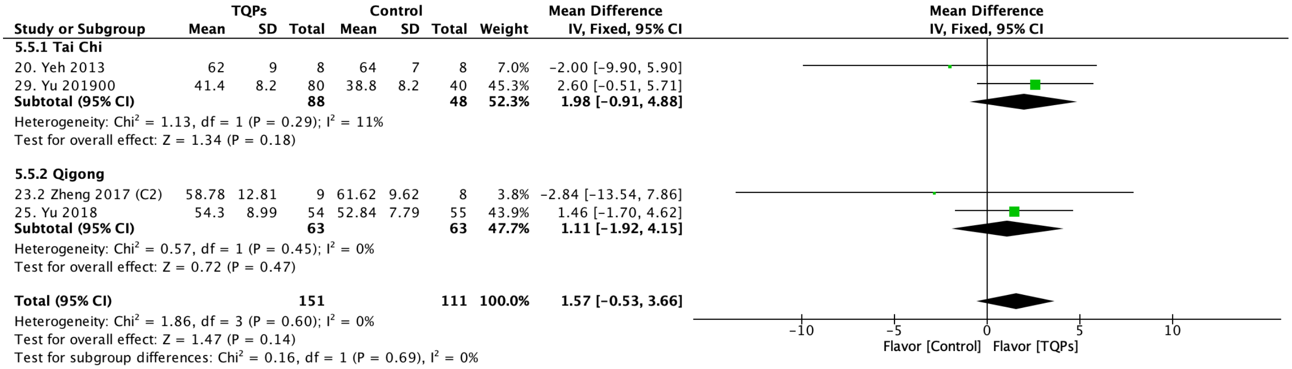

c. Subgroup analysis based on program durations

Study or Subgroup	TQPs Mean	TQPs SD	TQPs Total	Control Mean	Control SD	Control Total	Weight	Mean Difference IV, Fixed, 95% CI
**5.6.3 12 weeks**								
20. Yeh 2013	62	9	8	64	7	8	7.0%	−2.00 [−9.90, 5.90]
23.2 Zheng 2017 (C2)	58.78	12.81	9	61.62	9.62	8	3.8%	−2.84 [−13.54, 7.86]
25. Yu 2018	54.3	8.99	54	52.84	7.79	55	43.9%	1.46 [−1.70, 4.62]
**Subtotal (95% CI)**			**71**			**71**	**54.7%**	**0.72 [−2.11, 3.55]**
Heterogeneity: $Chi^2 = 1.09$, df = 2 (P = 0.58); $I^2 = 0\%$								
Test for overall effect: Z = 0.50 (P = 0.62)								
**5.6.5 24 weeks**								
29. Yu 201900	41.4	8.2	80	38.8	8.2	40	45.3%	2.60 [−0.51, 5.71]
**Subtotal (95% CI)**			**80**			**40**	**45.3%**	**2.60 [−0.51, 5.71]**
Heterogeneity: Not applicable								
Test for overall effect: Z = 1.64 (P = 0.10)								
**Total (95% CI)**			**151**			**111**	**100.0%**	**1.57 [−0.53, 3.66]**

Heterogeneity: $Chi^2 = 1.86$, df = 3 (P = 0.60); $I^2 = 0\%$
Test for overall effect: Z = 1.47 (P = 0.14)
Test for subgroup differences: $Chi^2 = 0.77$, df = 1 (P = 0.38), $I^2 = 0\%$

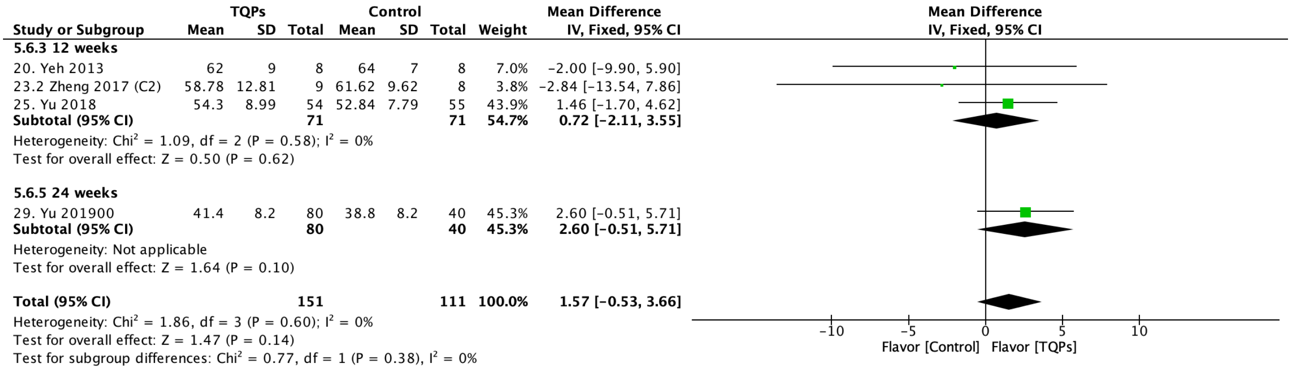

eFig. 10 BNP (pg/mL), TQPs plus RMs versus RMs (supplements to Fig. 6A)

a. The overall pooled results

Study or Subgroup	TQPs Mean	TQPs SD	TQPs Total	Control Mean	Control SD	Control Total	Weight	Mean Difference IV, Random, 95% CI
5. Huang 2014	255.97	99.2	44	256.15	95.68	44	15.2%	-0.18 [-40.90, 40.54]
6. Li 2015	215.3	79.9	90	272.4	87.9	90	16.0%	-57.10 [-81.64, -32.56]
8. Pan 2016	223	29	31	404	49	30	16.1%	-181.00 [-201.29, -160.71]
14. Yan 2016	249.51	229.34	36	382.16	279.52	36	9.9%	-132.65 [-250.76, -14.54]
15. Yang 2015	245.33	249.91	30	372.4	277.94	30	8.8%	-127.07 [-260.82, 6.68]
17. Yeh 2004	281	365	15	375	429	15	3.4%	-94.00 [-379.05, 191.05]
19. Yeh 2011	92	110.4	50	119	173.3	50	14.2%	-27.00 [-83.95, 29.95]
28. Li 2019	231.61	37.31	50	270.98	32.75	50	16.3%	-39.37 [-53.13, -25.61]
**Total (95% CI)**			**346**			**345**	**100.0%**	**-76.12 [-134.61, -17.62]**

Heterogeneity: Tau² = 5410.68; Chi² = 149.80, df = 7 (P < 0.00001); I² = 95%
Test for overall effect: Z = 2.55 (P = 0.01)

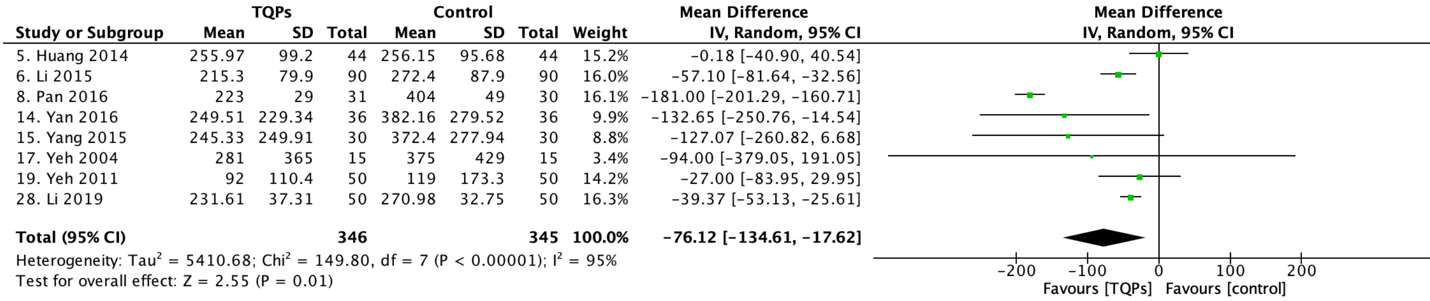

b. Subgroup analysis based on TQPs type

Study or Subgroup	TQPs Mean	TQPs SD	TQPs Total	Control Mean	Control SD	Control Total	Weight	Mean Difference IV, Random, 95% CI
**6.2.1 Tai Chi**								
5. Huang 2014	255.97	99.2	44	256.15	95.68	44	15.2%	-0.18 [-40.90, 40.54]
8. Pan 2016	223	29	31	404	49	30	16.1%	-181.00 [-201.29, -160.71]
17. Yeh 2004	281	365	15	375	429	15	3.4%	-94.00 [-379.05, 191.05]
19. Yeh 2011	92	110.4	50	119	173.3	50	14.2%	-27.00 [-83.95, 29.95]
**Subtotal (95% CI)**			**140**			**139**	**49.0%**	**-73.44 [-196.86, 49.98]**
Heterogeneity: Tau² = 13003.99; Chi² = 75.73, df = 3 (P < 0.00001); I² = 96%								
Test for overall effect: Z = 1.17 (P = 0.24)								
**6.2.2 Qigong**								
6. Li 2015	215.3	79.9	90	272.4	87.9	90	16.0%	-57.10 [-81.64, -32.56]
14. Yan 2016	249.51	229.34	36	382.16	279.52	36	9.9%	-132.65 [-250.76, -14.54]
15. Yang 2015	245.33	249.91	30	372.4	277.94	30	8.8%	-127.07 [-260.82, 6.68]
28. Li 2019	231.61	37.31	50	270.98	32.75	50	16.3%	-39.37 [-53.13, -25.61]
**Subtotal (95% CI)**			**206**			**206**	**51.0%**	**-52.10 [-75.21, -28.99]**
Heterogeneity: Tau² = 203.68; Chi² = 5.14, df = 3 (P = 0.16); I² = 42%								
Test for overall effect: Z = 4.42 (P < 0.00001)								
**Total (95% CI)**			**346**			**345**	**100.0%**	**-76.12 [-134.61, -17.62]**

Heterogeneity: Tau² = 5410.68; Chi² = 149.80, df = 7 (P < 0.00001); I² = 95%
Test for overall effect: Z = 2.55 (P = 0.01)
Test for subgroup differences: Chi² = 0.11, df = 1 (P = 0.74), I² = 0%

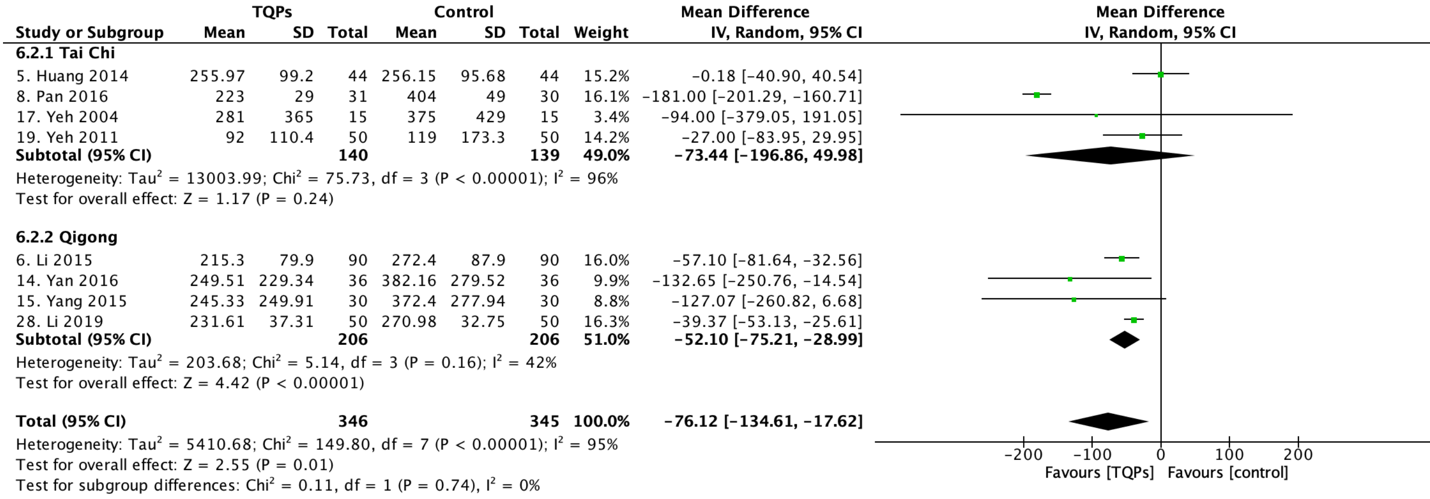

c. Subgroup analysis based on program durations

Study or Subgroup	TQPs Mean	TQPs SD	TQPs Total	Control Mean	Control SD	Control Total	Weight	Mean Difference IV, Random, 95% CI
**6.3.1 4 weeks**								
5. Huang 2014	255.97	99.2	44	256.15	95.68	44	15.2%	-0.18 [-40.90, 40.54]
**Subtotal (95% CI)**			**44**			**44**	**15.2%**	**-0.18 [-40.90, 40.54]**
Heterogeneity: Not applicable								
Test for overall effect: Z = 0.01 (P = 0.99)								
**6.3.3 12 weeks**								
17. Yeh 2004	281	365	15	375	429	15	3.4%	-94.00 [-379.05, 191.05]
19. Yeh 2011	92	110.4	50	119	173.3	50	14.2%	-27.00 [-83.95, 29.95]
28. Li 2019	231.61	37.31	50	270.98	32.75	50	16.3%	-39.37 [-53.13, -25.61]
**Subtotal (95% CI)**			**115**			**115**	**33.9%**	**-38.81 [-52.17, -25.45]**
Heterogeneity: Tau² = 0.00; Chi² = 0.32, df = 2 (P = 0.85); I² = 0%								
Test for overall effect: Z = 5.69 (P < 0.00001)								
**6.3.5 24 weeks**								
8. Pan 2016	223	29	31	404	49	30	16.1%	-181.00 [-201.29, -160.71]
**Subtotal (95% CI)**			**31**			**30**	**16.1%**	**-181.00 [-201.29, -160.71]**
Heterogeneity: Not applicable								
Test for overall effect: Z = 17.48 (P < 0.00001)								
**6.3.6 52 weeks**								
6. Li 2015	215.3	79.9	90	272.4	87.9	90	16.0%	-57.10 [-81.64, -32.56]
14. Yan 2016	249.51	229.34	36	382.16	279.52	36	9.9%	-132.65 [-250.76, -14.54]
15. Yang 2015	245.33	249.91	30	372.4	277.94	30	8.8%	-127.07 [-260.82, 6.68]
**Subtotal (95% CI)**			**156**			**156**	**34.7%**	**-72.87 [-116.33, -29.41]**
Heterogeneity: Tau² = 470.31; Chi² = 2.44, df = 2 (P = 0.30); I² = 18%								
Test for overall effect: Z = 3.29 (P = 0.001)								
**Total (95% CI)**			**346**			**345**	**100.0%**	**-76.12 [-134.61, -17.62]**

Heterogeneity: Tau² = 5410.68; Chi² = 149.80, df = 7 (P < 0.00001); I² = 95%
Test for overall effect: Z = 2.55 (P = 0.01)
Test for subgroup differences: Chi² = 145.98, df = 3 (P < 0.00001), I² = 97.9%

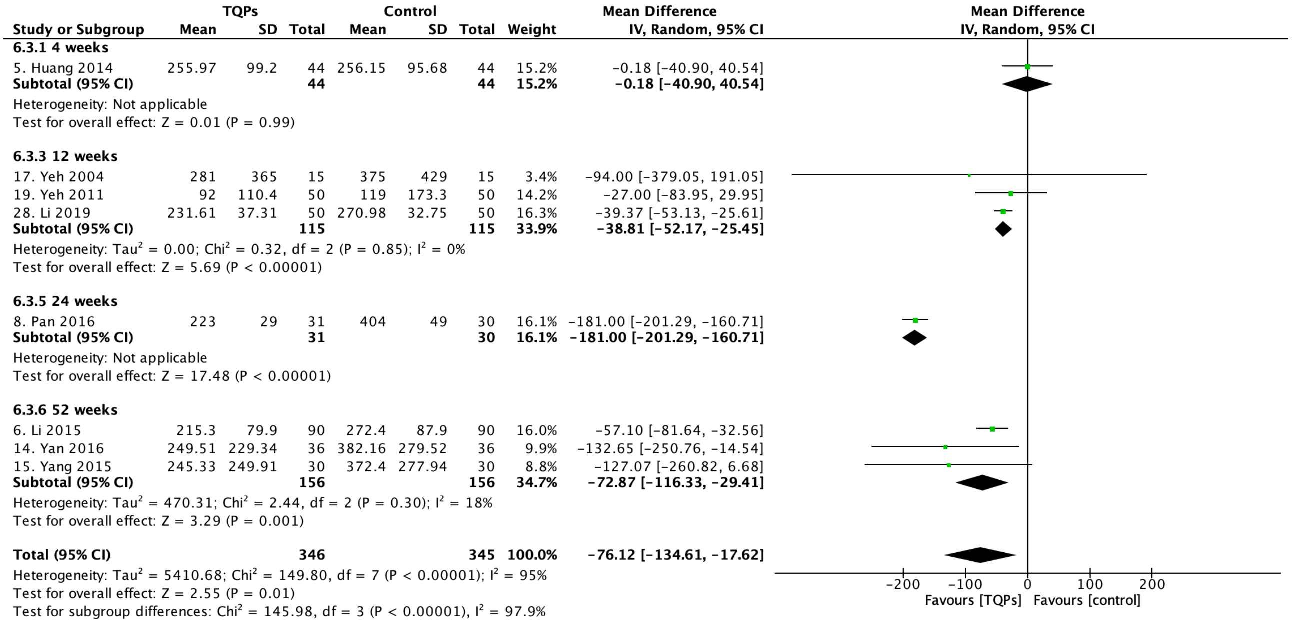

eFig. 11 BNP (pg/mL), TQPs plus RMs versus general exercises plus RMs (supplements to Fig. 6B)

a. The overall pooled results

Study or Subgroup	TQPs Mean	TQPs SD	TQPs Total	Control Mean	Control SD	Control Total	Weight	Mean Difference IV, Random, 95% CI
20. Yeh 2013	98	116	8	107	99	8	40.4%	-9.00 [-114.68, 96.68]
29. Yu 201900	300.2	80.6	80	191.2	80.6	40	59.6%	109.00 [78.41, 139.59]
**Total (95% CI)**			**88**			**48**	**100.0%**	**61.29 [-52.22, 174.79]**

Heterogeneity: $Tau^2 = 5386.63$; $Chi^2 = 4.42$, df = 1 (P = 0.04); $I^2 = 77\%$
Test for overall effect: Z = 1.06 (P = 0.29)

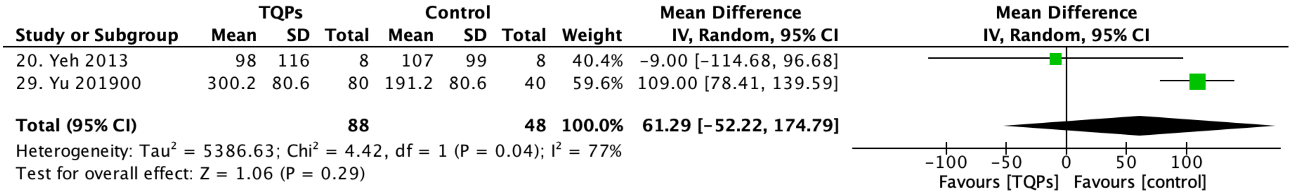

Supplementary Figures: Risk of bias and publication bias

eFig. 12 Risk of bias summary:

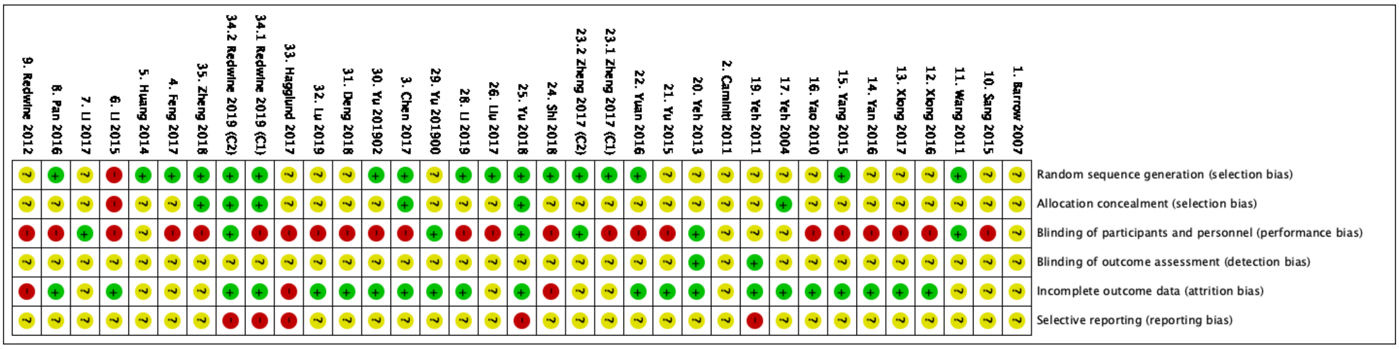

Legend: The authors' judgments about each risk of bias item for each included study

eFig. 13 Risk-of-bias graph

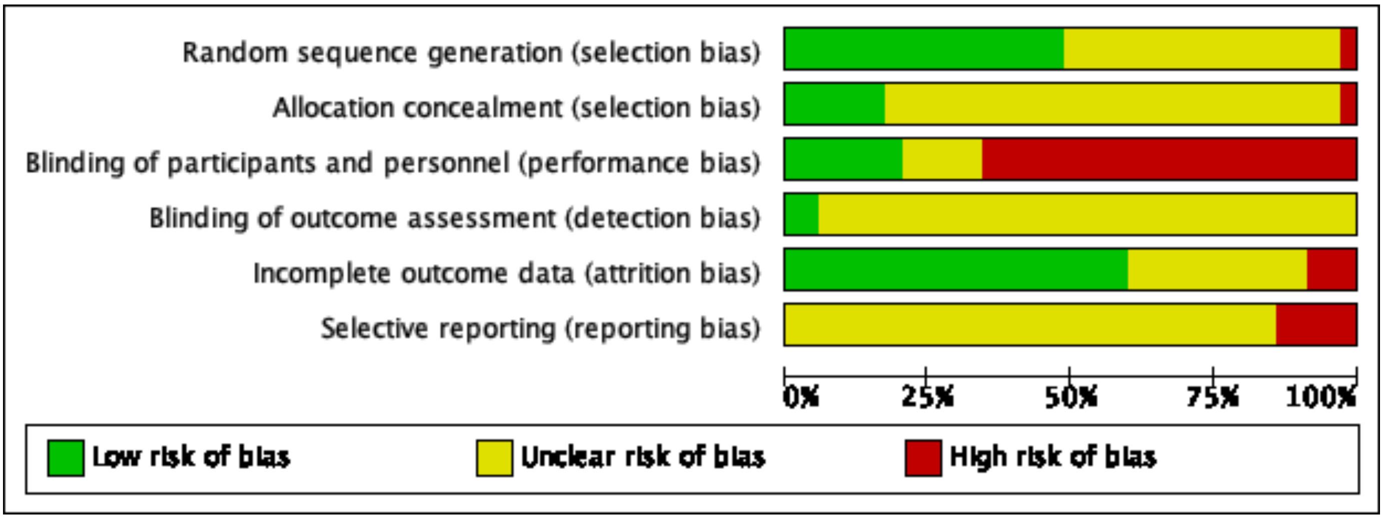

Legend: The authors' judgments regarding each risk-of-bias item presented as percentages across all included studies.

eFig. 14 Publication bias

a. 6MWD

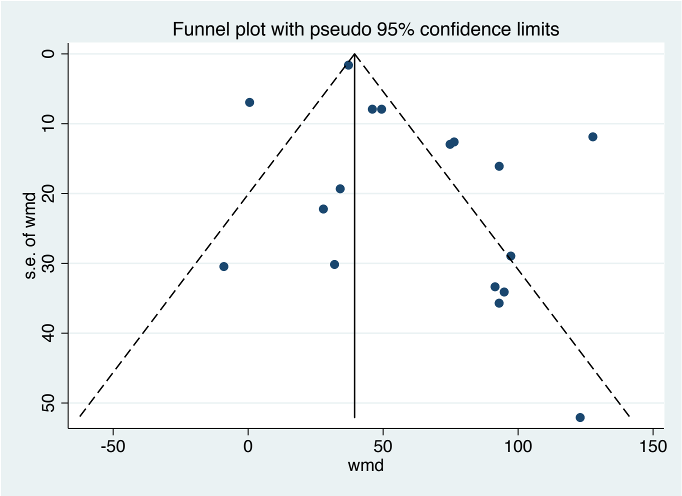

Legend: Egger's test: *P* = 0.079

b. MLHFQ

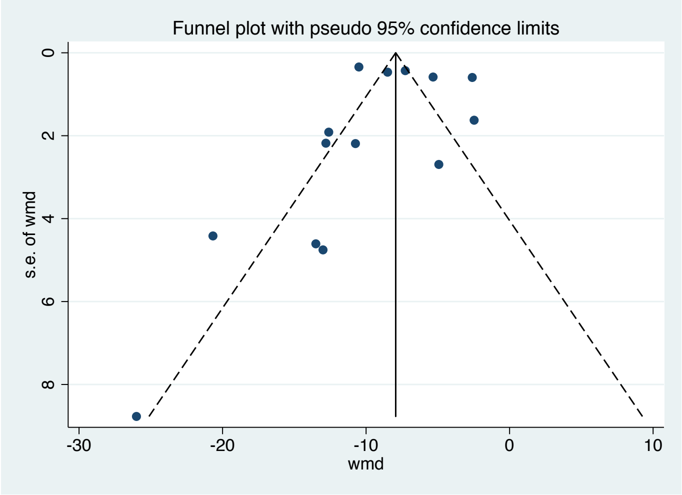

Legend: Egger's test: *P* = 0.817

c. LVEF (Egger's test: P = 0.929)

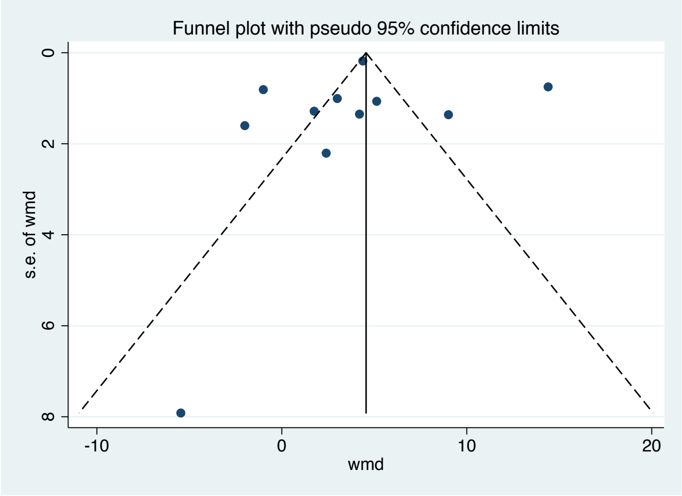

Legend: Egger's test: *P* = 0.929

PRISMA 2009 Checklist

Section/topic	#	Checklist item	Reported on page #
**TITLE**			
Title	1	Identify the report as a systematic review, meta-analysis, or both.	1
**ABSTRACT**			
Structured summary	2	Provide a structured summary including, as applicable: background; objectives; data sources; study eligibility criteria, participants, and interventions; study appraisal and synthesis methods; results; limitations; conclusions and implications of key findings; systematic review registration number.	2
**INTRODUCTION**			
Rationale	3	Describe the rationale for the review in the context of what is already known.	4-5
Objectives	4	Provide an explicit statement of questions being addressed with reference to participants, interventions, comparisons, outcomes, and study design (PICOS).	5
**METHODS**			
Protocol and registration	5	Indicate if a review protocol exists, if and where it can be accessed (e.g., Web address), and, if available, provide registration information including registration number.	5
Eligibility criteria	6	Specify study characteristics (e.g., PICOS, length of follow-up) and report characteristics (e.g., years considered, language, publication status) used as criteria for eligibility, giving rationale.	5-6
Information sources	7	Describe all information sources (e.g., databases with dates of coverage, contact with study authors to identify additional studies) in the search and date last searched.	5
Search	8	Present full electronic search strategy for at least one database, including any limits used, such that it could be repeated.	Supl
Study selection	9	State the process for selecting studies (i.e., screening, eligibility, included in systematic review, and, if applicable, included in the meta-analysis).	5-6
Data collection process	10	Describe method of data extraction from reports (e.g., piloted forms, independently, in duplicate) and any processes for obtaining and confirming data from investigators.	6-7
Data items	11	List and define all variables for which data were sought (e.g., PICOS, funding sources) and any assumptions and simplifications made.	6-7

Risk of bias in individual studies	12	Describe methods used for assessing risk of bias of individual studies (including specification of whether this was done at the study or outcome level), and how this information is to be used in any data synthesis.	7
Summary measures	13	State the principal summary measures (e.g., risk ratio, difference in means).	7-8
Synthesis of results	14	Describe the methods of handling data and combining results of studies, if done, including measures of consistency (e.g., I^2) for each meta-analysis.	7-8

Section/topic	#	Checklist item	Reported on page #
Risk of bias across studies	15	Specify any assessment of risk of bias that may affect the cumulative evidence (e.g., publication bias, selective reporting within studies).	8
Additional analyses	16	Describe methods of additional analyses (e.g., sensitivity or subgroup analyses, meta-regression), if done, indicating which were pre-specified.	8
**RESULTS**			
Study selection	17	Give numbers of studies screened, assessed for eligibility, and included in the review, with reasons for exclusions at each stage, ideally with a flow diagram.	9
Study characteristics	18	For each study, present characteristics for which data were extracted (e.g., study size, PICOS, follow-up period) and provide the citations.	9-10
Risk of bias within studies	19	Present data on risk of bias of each study and, if available, any outcome level assessment (see item 12).	12
Results of individual studies	20	For all outcomes considered (benefits or harms), present, for each study: (a) simple summary data for each intervention group (b) effect estimates and confidence intervals, ideally with a forest plot.	10-12
Synthesis of results	21	Present results of each meta-analysis done, including confidence intervals and measures of consistency.	Figure 1
Risk of bias across studies	22	Present results of any assessment of risk of bias across studies (see Item 15).	12
Additional analysis	23	Give results of additional analyses, if done (e.g., sensitivity or subgroup analyses, meta-regression [see Item 16]).	12
**DISCUSSION**			
Summary of evidence	24	Summarize the main findings including the strength of evidence for each main outcome; consider their relevance to key groups (e.g., healthcare providers, users, and policy makers).	13
Limitations	25	Discuss limitations at study and outcome level (e.g., risk of bias), and at review-level (e.g., incomplete retrieval of identified research, reporting bias).	15
Conclusions	26	Provide a general interpretation of the results in the context of other evidence, and implications for future research.	16
**FUNDING**			

Funding	27	Describe sources of funding for the systematic review and other support (e.g., supply of data); role of funders for the systematic review.	17

From: Moher D, Liberati A, Tetzlaff J, Altman DG, The PRISMA Group (2009). Preferred Reporting Items for Systematic Reviews and Meta-Analyses: The PRISMA Statement. PLoS Med 6(6): e1000097. doi:10.1371/journal.pmed1000097

For more information, visit: **www.prisma-statement.org**.
